# Further validation of strecker-type α-aminonitriles as a new class of potent human carbonic anhydrase II inhibitors: hit expansion within the public domain using differential scanning fluorimetry leads to chemotype refinement

**DOI:** 10.1080/14756366.2019.1693556

**Published:** 2019-11-22

**Authors:** Mikhail Krasavin, Stanislav Kalinin, Sergey Zozulya, Anastasia Griniukova, Petro Borysko, Andrea Angeli, Claudiu T. Supuran

**Affiliations:** a Saint Petersburg State University, Saint Petersburg, Russian Federation; b Enamine Ltd, Kyiv, Ukraine; c Taras Shevchenko National University, Kyiv, Ukraine; d Neurofarba Department, Universita degli Studi di Firenze, Florence, Italy

**Keywords:** Differential scanning fluorimetry, thermal shift assay, protein affinity, carbonic anhydrase II

## Abstract

Testing of an expanded, 800-compound set of analogues of the earlier described Strecker-type α-aminonitriles (selected from publicly available Enamine Ltd. Screening Collection) in thermal shift assay against bovine carbonic anhydrase (*b*CA) led to further validation of this new class of inhibitors and identification a new, refined chemotype represented by inhibitors with 10-improved potency. 
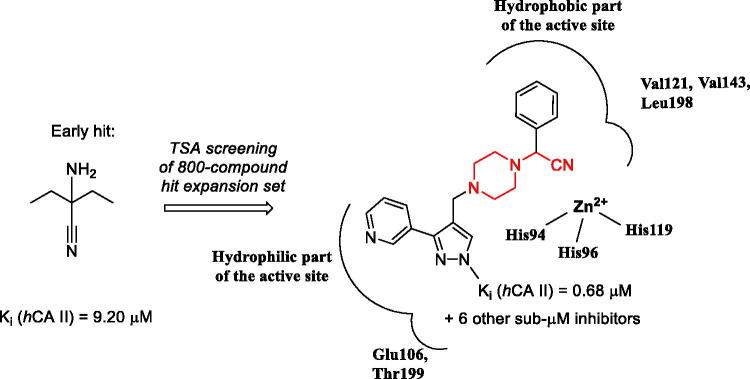

## Introduction

**Figure 1.**
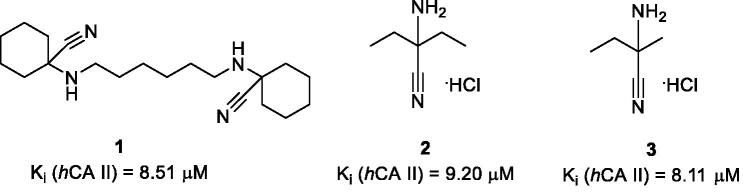
Strecker α-aminonitrile *h*CA II inhibitory hits **1–3** discovered earlier *via* the DSF screening.

Differential scanning fluorimetry (DSF), also termed thermal shift assay (TSA), is an efficient technique for direct determination of a small molecule’s affinity to a protein target^1^. The underlying principle of the method is the ability of a small molecule binding to the protein, in principle, to stabilise or destabilise the tertiary structure of the macromolecule and thus increase or decrease its melting temperature (*T_m_*), respectively. Earlier, we conducted a high-throughput DSF screening of a chemically diverse set of 8000 compounds selected from the Enamine screening collection comprising over 2,000,000 compounds^2^ against bovine carbonic anhydrase (*b*CA), a protein most closely resembling isoform II of the human carbonic anhydrase (*h*CA II). This led to the discovery of three compounds (**1–3**) that produced noticeable thermal shift of *b*CA *T_m_* (Δ*T_m_*) which did not belong to any of then-known classes of carbonic anhydrase inhibitors and which we dubbed as Strecker α-aminonitriles considering they could be obtained from various ketones *via* the Strecker reaction. Besides the TSA biophysical readout, these compounds were found, by testing in biochemical stopped-flow kinetics CO_2_ hydration assay, to inhibit *h*CA II (for which *b*CA was initially selected as a surrogate protein) in the single-digit micromolar range (Figure 1)^3^.

As it was confirmed that compounds **1–3** acted as true inhibitors (and sources of adventitious cyanide anion which is known to inhibit *h*CA), we postulated that α-aminonitriles in general may act as suicide *in situ* donors of CN^-^ anion (similarly to N-cyanosulfonamides reported by Supuran et al.^4^) and thus *h*CA inhibition in general (and *h*CA II in particular) can be expected from any representatives of this chemotype with sufficient complementarity (affinity) to the enzyme’s active site. In order to verify this hypothesis and also to discover more potent inhibitors belonging to this class of compounds as well as to establish structure-activity relationships (SAR), we undertook more focussed screening of 800 publicly available α-aminonitriles selected from the Enamine Ltd. screening collection^2^. This led, after subsequent biochemical testing of the strongest ‘thermal shifters’, to the discovery of a series of submicromolar inhibitors of *h*CA II, an established target for glaucoma and diuretic drugs^5^. Herein, we present the results obtained in the course of these efforts.

## Materials and methods

### Chemical compounds

The 800 α-aminonitriles (for the full list, see Supplemental Material) for the follow-up DSF screening were selected by substructure search and obtained directly from the Enamine screening collection^2^. Their identity and purity was confirmed by ^1^H NMR spectroscopy prior to biochemical testing in CA inhibition assay.

### Differential scanning fluorimetry (thermal shift) assay

Thermal shift assay was carried out using ViiA™ 7 Real-Time PCR System equipped with 384-well block (Applied Biosystems, USA). The TSA procedure was adopted from the literature^6–8^ and was modified in order to allow measuring the *b*CA melting temperature on interaction with various compounds, including the known CA inhibitor azetazolamide (AZ)^9^, which was used in this study as a reference *b*CA binder at 10 and 20 µM concentration.

For 800-compound screening, the test reactions were set up in the following buffer: 10 mM NaH_2_PO_4_/Na_2_HPO_4_, pH 7.0, 10 mM NaCl. The total volume of the reaction mixture per well was 10 µL. Carbonic anhydrase (Sigma Aldrich Cat# C3934) in 300 µg/mL concentration was pre-mixed with environment-sensitive SYPRO orange dye (Invitrogen, Cat# S6650) at final concentration in the reaction of 10 ×, with regard to the stock concentration stated by the vendor. The mixtures were pre-incubated for 1 h at 4 °C with 20 µM concentrations of the compounds (and 1% final concentration of DMSO), placed into MicroAmp^®^ Optical 384-Well Reaction Plate (ThermoFisher, Cat# 4309849). The reaction mixture was kept at room temperature for 15 min to ensure full protein-compound interactions. The temperature was raised at 1.6 °C/s rate to 40 °C without signal reading. Starting from 40 °C up to 85 °C the heating rate was set to 0.05 °C/s with constant fluorescence reading, using 470/623 nm filter set. The raw data of dye fluorescence intensity change upon melting of the protein were obtained from the instrument ViiA 7 RUO software. Further data processing and visualisation was performed by custom-made Microsoft Excel scripts. The peak of the first derivative for the fluorescence curve was used to define melting temperature *T_m_*. *T_m_* for DMSO control wells, having only protein, dye and 1% DMSO was used as a *T_o_* to determine melting temperature shifts (Δ*T_m_*). All measurements were made in quadruplicates.

### Carbonic anhydrase inhibition assay

An Applied Photophysics stopped-flow instrument was used for assaying the CA catalysed CO_2_ hydration activity^10^. Phenol red (at a concentration of 0.2 mM) was used as an indicator, working at the absorbance maximum of 557 nm, with 20 mM Hepes (pH 7.5) as a buffer, and 20 mM Na_2_SO_4_ (for maintaining constant ionic strength), following the initial rates of the CA-catalysed CO_2_ hydration reaction for a period of 10–100 s. The CO_2_ concentrations ranged from 1.7 to 17 mM for the determination of the kinetic parameters and inhibition constants. For each inhibitor at least six traces of the initial 5–10% of the reaction have been used for determining the initial velocity. The non-catalysed rates were determined in the same manner and subtracted from the total observed rates. Stock solutions of inhibitor (0.1 mM) were prepared in distilled-deionized water and dilutions up to 0.01 nM were done thereafter with the assay buffer. Inhibitor and enzyme solutions were combined and pre-incubated for 15 min at room temperature prior to running the assay, in order to allow for the formation of the ezyme–inhibitor complex. The inhibition constants were obtained by non-linear least-squares methods using PRISM 3 and the Cheng-Prusoff equation, as reported earlier^11^, and represent the mean from at least three different determinations. Recombinant *h*CA II was obtained in-house as reported earlier^12–16^.

## Results and discussion

Considering that amino acetonitrile moiety (characteristic of the Strecker-type α-aminonitrile chemotype) was postulated as the pharmacophore for the recently discovered^3^ class of carbonic anhydrase inhibitors, we selected 800 compounds featuring this motif (see Supplemental Data) from the Enamine, Ltd. Screening Collection^2^ and screened this set at 20 µM concentration against *b*CA for the ability to shift the enzyme’s melting temperature (*T_m_*) using acetazolamide (AZ)^9^ as the positive comparator (producing a > 5 °C shift in *b*CA *T_m_*^3^).

**Figure 2.**
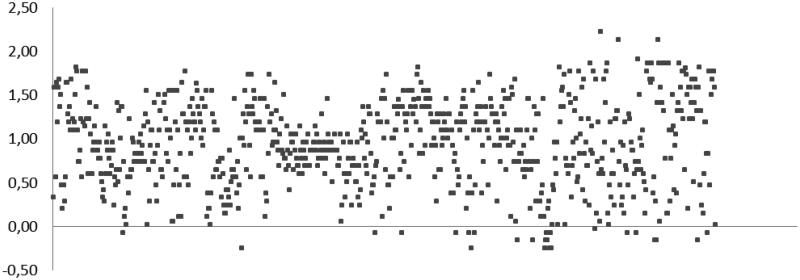
Thermal shift (Δ*T_m_*, °C) for the 800 α-aminonitriles screened in DSF assay.

As it follows from the graphical representation of the TSA screening data obtained for the 800 compounds (Figure 2), none of the compounds produced an equally strong thermal shift compared to AZ. However, quite a few compounds (precisely, 375) populated the >1.0 °C area. In order to select compounds from this subset of ‘stronger shifters’ for subsequent biochemical testing of carbonic anhydrase inhibition, the value of the thermal shift (Δ*T_m_*_,_°C) as well as the quality of the melting curve obtained (evaluated by visual inspection) were taken into account. With these two criteria, a total of 47 compounds were selected and tested in stopped-flow CO_2_ hydration assay for their ability to inhibit *h*CA II. The *K_i_* values presented in Table 1 clearly demonstrate that for a substantial number of compounds, the effect on the enzyme’s melting point determined by TSA, did not translate into potent (or any) inhibition of *h*CA II.

**Table 1.** *h*CA II inhibition (*K_i_*) data of 48 compounds selected from the initial 800-compound screening set based on their *b*CA thermal shift (Δ*T_m_*, °C) values.

Compound	Enamine ID^2^	Structure	Δ*T_m_*, °C	*K_i_* (μM)
**4**	Z118259550		1.23	>100
**5**	Z131022682		1.77	8.1
**6**	Z123607262		1.32	89.3
**7**	Z118259780		1.14	>100
**8**	Z118260178		1.14	74.3
**9**	Z118259626		1.23	>100
**10**	Z146786712		1.50	53.7
**11**	Z1126852296		1.59	9.1
**12**	Z913897024		1.77	40.9
**13**	Z1097120891		1.59	7.4
**14**	Z1230758078		1.50	9.3
**15**	Z1251205230		1.41	>100
**16**	Z1251205249		1.50	0.97
**17**	Z1176496598		1.14	0.78
**18**	Z1185260979		1.41	0.93
**19**	Z1204316164		1.23	9.6
**20**	Z1204317166		1.01	100
**21**	Z1205181012		1.55	>100
**22**	Z1261078980		1.55	>100
**23**	Z1322755479		1.55	9.1
**24**	Z1298330150		1.55	16.0
**25**	Z1298979831		1.64	36.1
**26**	Z1297989190		1.55	3.0
**27**	Z1343956502		1.46	>100
**28**	Z1317906129		1.46	8.9
**29**	Z1317905741		1.55	3.7
**30**	Z146384300		1.64	0.91
**31**	Z1390085782		1.55	0.76
**32**	Z1395303258		1.73	0.86
**33**	Z1395304494		1.73	5.3
**34**	Z1567293695		1.73	5.0
**35**	Z1171402445		1.82	60.5
**36**	Z1167646026		1.64	29.8
**37**	Z1395305944		1.46	2.3
**38**	Z1567199718		1.28	5.0
**39**	Z1167645477		1.64	20.4
**40**	Z1124717499		1.64	41.9
**41**	Z1416783341		1.73	35.1
**42**	Z1198235528		1.01	>100
**43**	Z1198234963		0.65	>100
**44**	Z118259850		1.37	15.7
**45**	Z1567277098		1.55	40.9
**46**	Z1126852482		1.37	3.7
**47**	Z1176495724		1.19	38.2
**48**	Z1567280757		1.46	5.5
**49**	Z1317905463		1.63	3.5
**50**	Z1470771430		1.37	0.68
AZ			>5.0	0.012

*Mean *K_i_* values from three different stopped-flow assays (errors were in the range of ±5–10% of the reported values).

**Figure 3.**
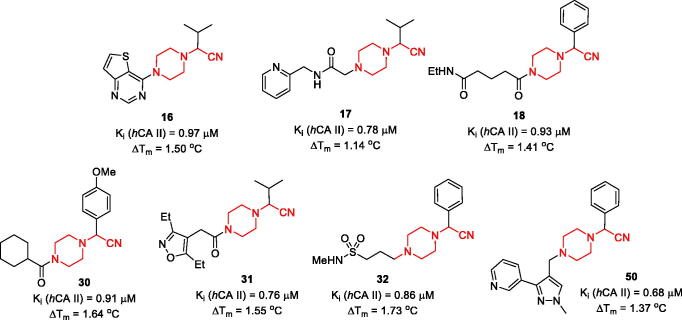
Most potent, *N*-(cyanomethyl)piperazine *h*CA II inhibitors discovered in the course of this study.

A substantial number of TSA hits displayed biochemical *h*CA II inhibition in the same single-digit micromolar *K_i_* range as the initial hits (**1–3**). Reassuringly, however, seven compounds displayed *K_i_* values in the submicromolar range, which represented a 10-fold improvement compared to inhibitory potency of **1–3** towards *h*CA II. Moreover, these compounds, while belonging to the general Strecker α-aminonitrile class, turned out to be structurally distinct from the initial hits 1–3 as they all are based on a new *N*-(cyanomethyl)piperazine scaffold (Figure 3).

**Figure 4.**
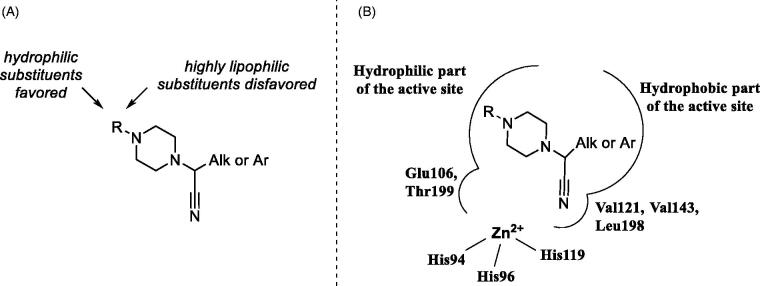
Preliminary SAR generalisations (A) and possible binding mode of *N*-(cyanomethyl)piperazines.

It is also evident that while all frontrunner compounds (**16**–**18**, **30**–**32** and **50**) bear a hydrophobic aliphatic or aromatic substituent on the same carbon atom as the cyano group, the *N^4^* position of the piperazine ring is substituted with a relatively polar group. Moreover, examination of the SAR information contained in the total TSA screening data (Supplemental Information), reveals that hydrophobic groups at *N^4^* reduce the compound’s affinity to the target (as indirectly measured by Δ*T_m_*). Such an active chemotype topology appear to be in line with the known distinct architecture of the carbonic anhydrase active site where two halves – hydrophobic and hydrophilic are clearly delineated^5^. Considering the fact that in order to exert its inhibitory potency (hypothesized to include suicide donation of the cyanide anion *in situ*^3^), the inhibitor’s molecule must have complementarity to its active site, such hydrophilic/hydrophobic dichotomy of the most active inhibitors matches that of the hCA II active site (Figure 4).

## Conclusion

Testing of an expanded, 800-compound set of analogues of the earlier described Strecker-type α-aminonitriles in thermal shift assay against bovine carbonic anhydrase (*b*CA) led not only to further validation of this new class of inhibitors but also to refinement of the active chemotype, which became possible after biochemical testing of 47 selected TSA hits for *h*CA II inhibition in stopped-flow CO_2_ hydration assay. The active chemotype can be defined as *N^1^*-(cyanomethyl)piperazine bearing two other substituents (hydrophilic and hydrophobic) on the opposite sides of the piperazine core. Such a dichotomy of the newly identified pharmacophore appears to be in line with the known bipolar character of the enzyme’s active site. Further studies are underway to investigate the validity of this hypothesis.

## Supplementary Material

Supplemental Material
