# Supplementary material for: Further validation of strecker-type α-aminonitriles as a new class of potent human carbonic anhydrase II inhibitors: hit expansion within the public domain using differential scanning fluorimetry leads to chemotype refinement
Source: J Enzyme Inhib Med Chem. 2019 Nov 22;35(1):165–71. doi: 10.1080/14756366.2019.1693556 (PMC6882446; doi:10.1080/14756366.2019.1693556)
Supplement: Supplemental Material [file IENZ_A_1693556_SM9305.pdf]

Supplemental Material for

Further validation of Strecker-type α-aminonitriles as a new class of potent human carbonic anhydrase II inhibitors: hit expansion within the public domain using differential scanning fluorimetry leads to chemotype refinement

Mikhail Krasavin, * Stanislav Kalinin, Sergey Zozulya, Petro Borysko, Andrea Angeli, Claudiu T. Supuran *

Contents:

Results of the thermal shift screening of the 800-compound set against *b*CA pp. 2-116

Entry	Structure	ID	Tm D	delta TmD	Curve reliability	Selected for biochemical assay?
1	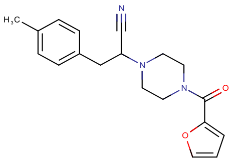	Z103729778	67,12	0,33		
2	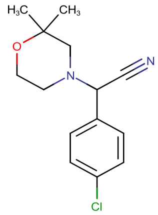	Z1097120891	68,38	1,59	++	YES
3	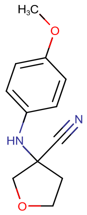	Z1124555255	67,29	0,56		
4	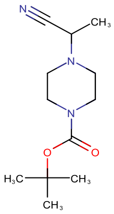	Z1124717499	68,35	1,64	++	YES
5	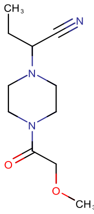	Z1124717695	67,92	1,19	++	
6	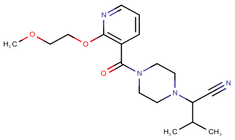	Z1126852296	68,38	1,59	++	YES
7	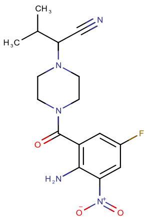	Z1126852433	68,47	1,68	++	

8	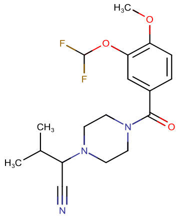	Z1126852482	68,08	1,37	++	YES
9	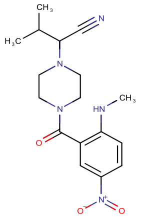	Z1126852649	68,29	1,50	++	
10	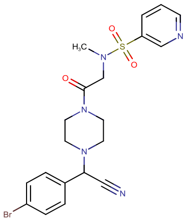	Z1126993651	67,18	0,47		
11	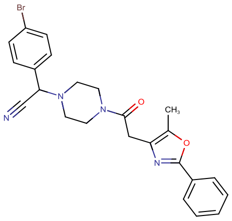	Z1126993754	66,91	0,20		
12	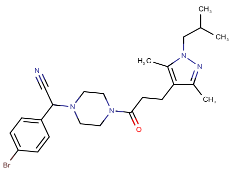	Z1126993887	67,18	0,47		
13	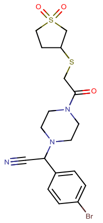	Z1126993942	67,18	0,47		
14	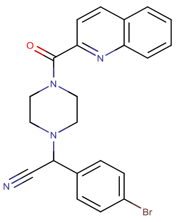	Z1126994076	67,00	0,29		

15	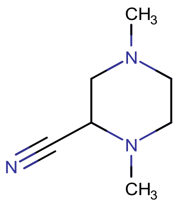	Z1162448516	67,29	0,56		
16	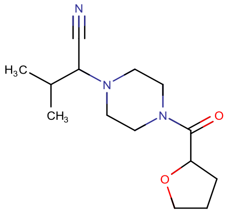	Z1167645477	68,35	1,64	++	YES
17	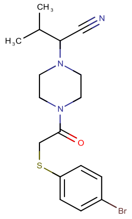	Z1167646026	68,35	1,64	++	YES
18	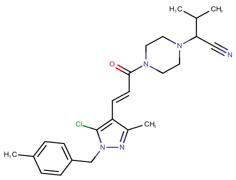	Z1167646457	67,99	1,28	++	
19	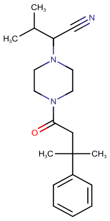	Z1167647281	67,92	1,19	++	
20	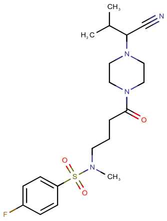	Z1167647565	68,08	1,37	++	
21	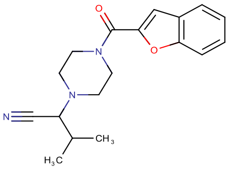	Z1167648709	67,83	1,10	++	

22	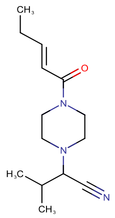	Z1168717314	68,47	1,68	++	
23	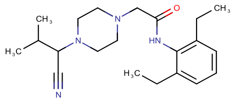	Z1171401571	67,83	1,10	++	
24	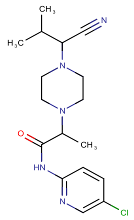	Z1171401737	68,02	1,23	++	
25	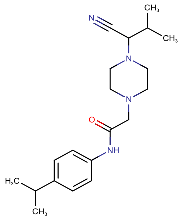	Z1171401767	67,83	1,10	++	
26	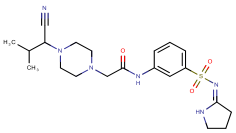	Z1171402368	67,90	1,19	++	
27	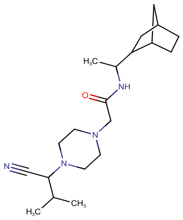	Z1171402412	68,29	1,50	++	
28	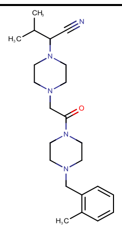	Z1171402445	68,53	1,82	++	YES

29	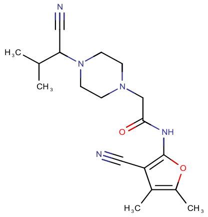	Z1171402602	68,56	1,77	++	
30	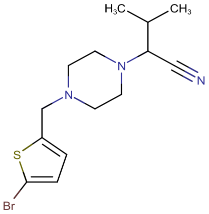	Z1171403104	67,81	1,10	++	
31	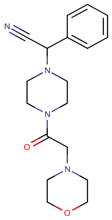	Z1171447839	67,47	0,74		
32	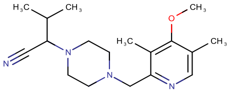	Z1171978412	67,47	0,74		
33	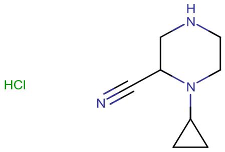	Z1171980563	67,66	0,87		
34	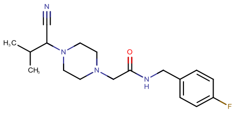	Z1171980643	68,02	1,23	++	
35	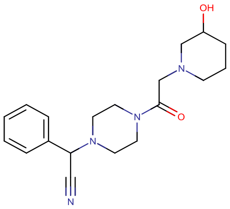	Z1176191421	67,29	0,56		

36	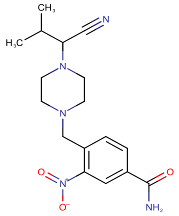	Z1176495643	68,56	1,77	++	
37	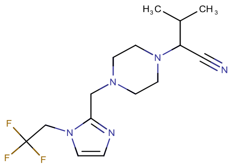	Z1176495724	67,92	1,19	++	YES
38	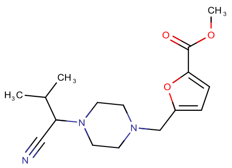	Z1176496546	68,56	1,77	++	
39	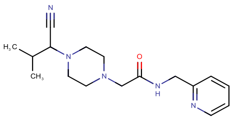	Z1176496598	67,93	1,14	++	YES
40	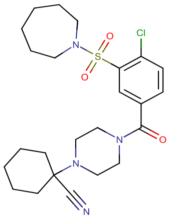	Z118259252	68,56	1,77	++	
41	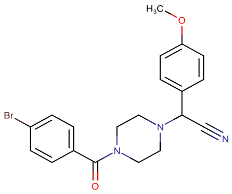	Z118259550	68,02	1,23	++	YES
42	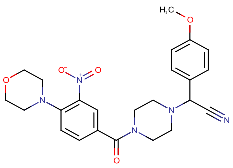	Z118259552	68,38	1,59	++	

43	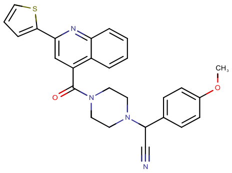	Z118259604	67,39	0,60		
44	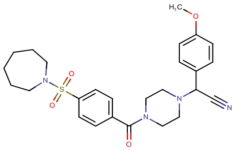	Z118259626	68,02	1,23	++	YES
45	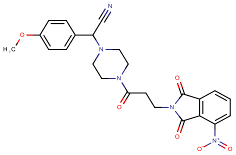	Z118259700	68,20	1,41	++	
46	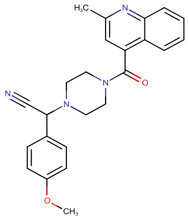	Z118259706	67,84	1,05	++	
47	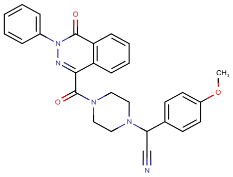	Z118259712	67,39	0,60		
48	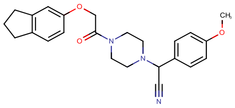	Z118259750	67,66	0,87		
49	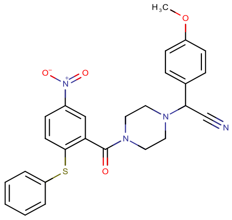	Z118259752	67,75	0,96		

50	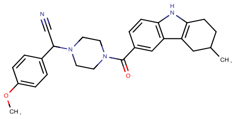	Z118259780	67,93	1,14	++	YES
51	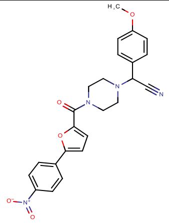	Z118259848	67,75	0,96		
52	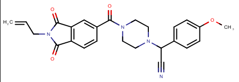	Z118259850	68,08	1,37	++	YES
53	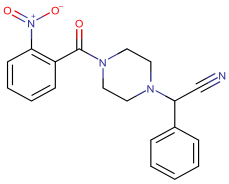	Z118259880	67,66	0,87		
54	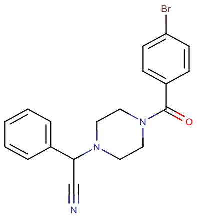	Z118259890	67,66	0,87		
55	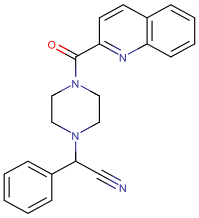	Z118259894	67,57	0,78		
56	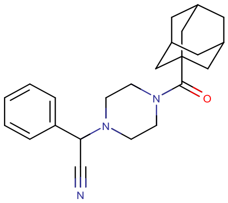	Z118259908	67,75	0,96		

57	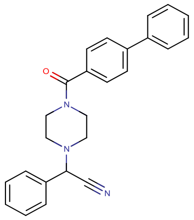	Z118259930	67,57	0,78		
58	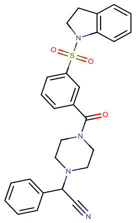	Z118259950	67,39	0,60		
59	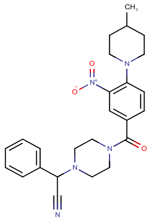	Z118260000	67,30	0,51		
60	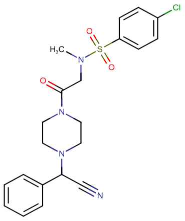	Z118260050	67,12	0,33		
61	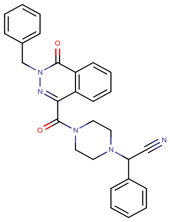	Z118260058	67,21	0,42		
62	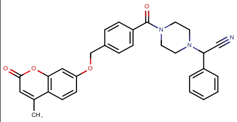	Z118260084	67,00	0,29		
63	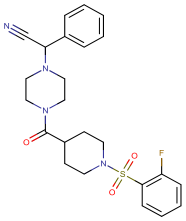	Z118260178	67,93	1,14	++	YES

64	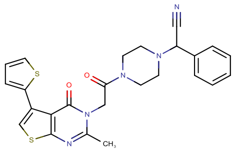	Z118260228	67,39	0,60		
65	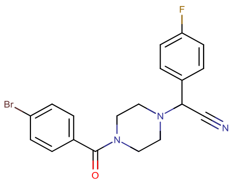	Z118260242	67,75	0,96		
66	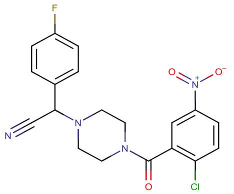	Z118260248	67,66	0,87		
67	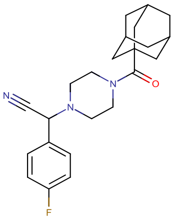	Z118260260	67,84	1,05	++	
68	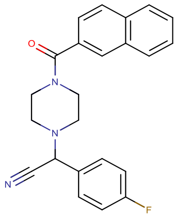	Z118260266	67,57	0,78		
69	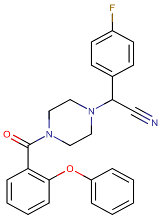	Z118260288	67,39	0,60		
70	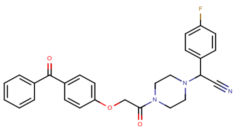	Z118260366	67,66	0,87		

71	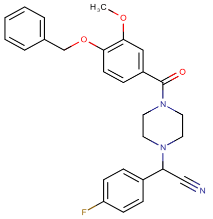	Z118260420	67,48	0,69		
72	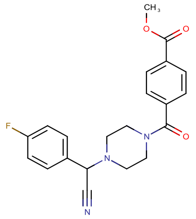	Z118260450	67,75	0,96		
73	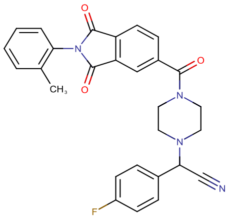	Z118260466	67,66	0,87		
74	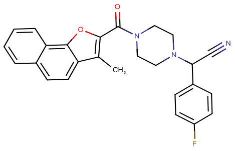	Z118260486	67,03	0,24		
75	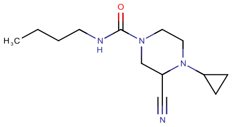	Z1183988703	67,27	0,56		
76	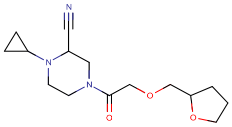	Z1184198603	67,20	0,47		
77	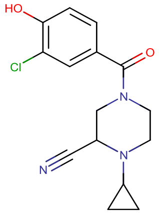	Z1184202249	67,36	0,65		

78	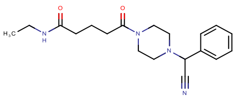	Z1185260979	68,20	1,41	++	YES
79	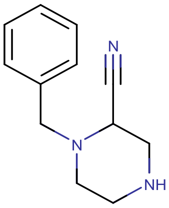	Z1197875434	67,30	0,51		
80	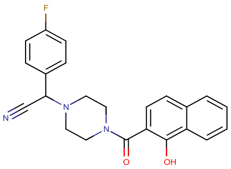	Z119802104	68,11	1,32	++	
81	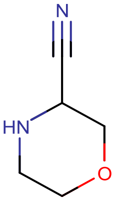	Z1198234963	67,36	0,65		YES
82	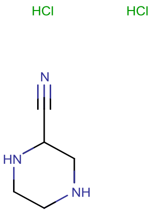	Z1198235528	67,72	1,01	++	YES
83	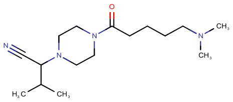	Z1203557852	68,08	1,37	++	
84	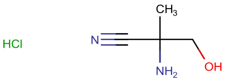	Z1203579706	68,08	1,37	++	

85	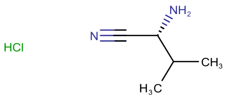	Z1203581086	66,64	-0,07		
86	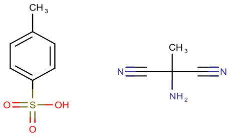	Z1203581232	67,45	0,74		
87	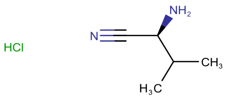	Z1203582231	66,82	0,11		
88	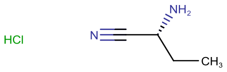	Z1203638467	66,91	0,20		
89	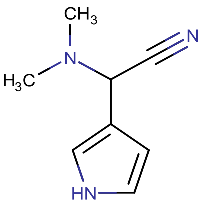	Z1203746605	66,73	0,02		
90	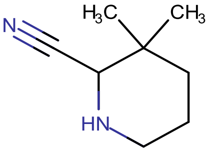	Z1203747529	67,29	0,56		
91	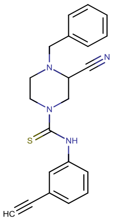	Z1203912801	67,09	0,38		

92	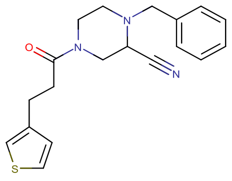	Z1204316164	68,02	1,23	++	YES
93	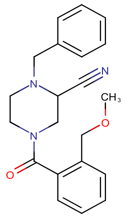	Z1204316493	67,29	0,56		
94	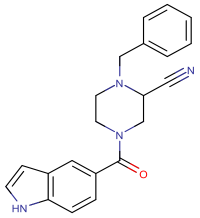	Z1204316904	67,36	0,65		
95	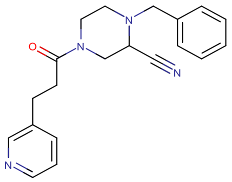	Z1204317109	67,38	0,65		
96	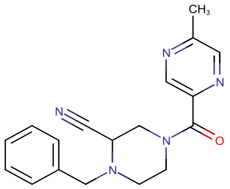	Z1204317166	67,72	1,01	++	YES
97	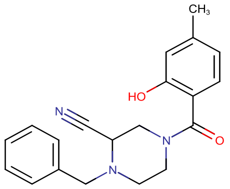	Z1204317223	67,75	0,96		
98	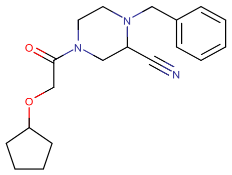	Z1204317431	67,47	0,74		

99	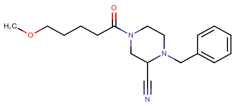	Z1204317452	67,56	0,83		
100	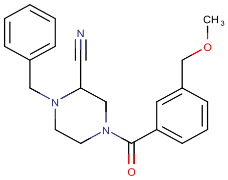	Z1204317599	67,54	0,83		
101	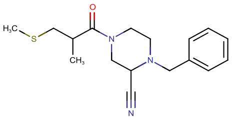	Z1204317779	67,56	0,83		
102	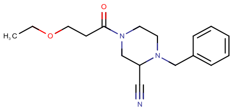	Z1204317819	67,47	0,74		
103	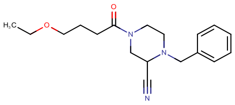	Z1204317981	67,54	0,83		
104	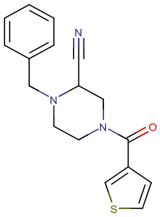	Z1204318067	67,75	0,96		
105	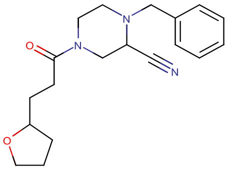	Z1204318481	67,45	0,74		

106	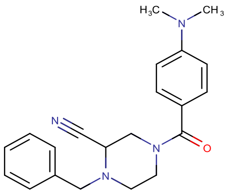	Z1204318673	67,54	0,83		
107	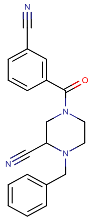	Z1204318678	67,63	0,92		
108	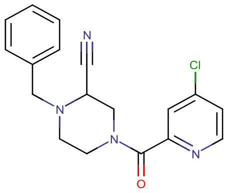	Z1204318970	67,47	0,74		
109	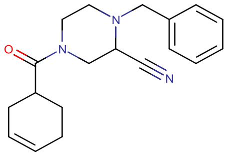	Z1204319117	67,93	1,14	++	
110	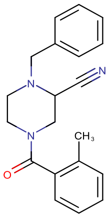	Z1204319142	67,47	0,74		
111	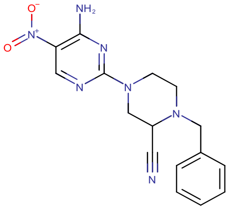	Z1205180878	67,99	1,28	++	
112	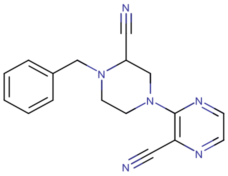	Z1205181012	68,26	1,55	++	YES

113	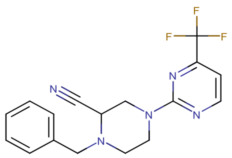	Z1205181089	66,73	0,02		
114	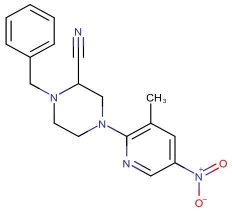	Z1205181248	67,90	1,19	++	
115	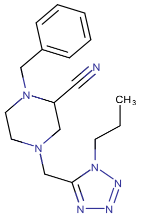	Z1205416243	67,36	0,65		
116	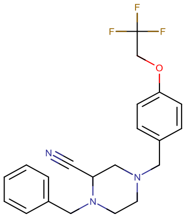	Z1205416507	67,00	0,29		
117	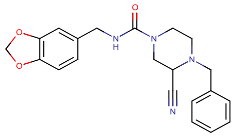	Z1205510466	67,63	0,92		
118	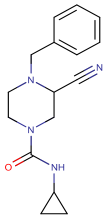	Z1206397866	67,81	1,10	++	
119	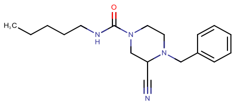	Z1206397868	67,81	1,10	++	

120	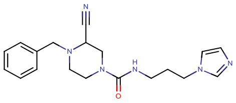	Z1206397869	67,47	0,74		
121	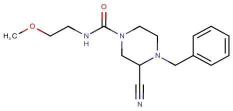	Z1206397876	67,45	0,74		
122	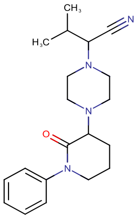	Z1212824601	68,20	1,41	++	
123	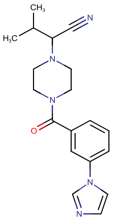	Z1225083562	67,56	0,83		
124	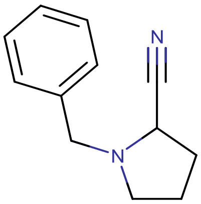	Z1227729368	67,20	0,47		
125	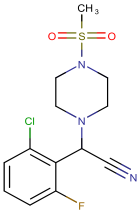	Z1230750087	67,00	0,29		
126	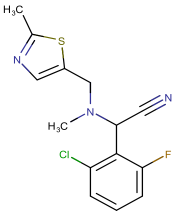	Z1230755319	67,65	0,92		

127	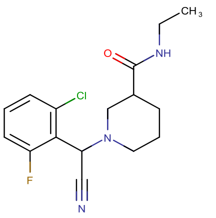	Z1230758078	68,29	1,50	++	YES
128	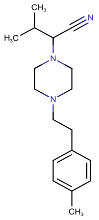	Z1232771840	67,81	1,10	++	
129	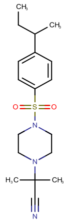	Z123607262	68,11	1,32	++	YES
130	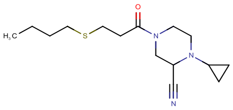	Z1246097017	67,38	0,65		
131	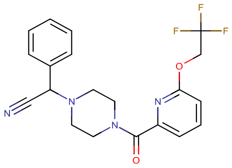	Z1246440832	67,36	0,65		
132	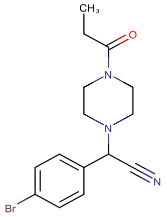	Z1250628642	67,27	0,56		
133	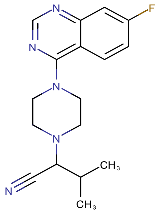	Z1251205230	68,20	1,41	++	YES

134	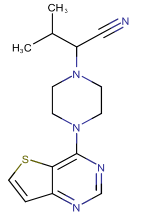	Z1251205249	68,29	1,50	++	YES
135	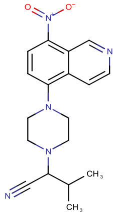	Z1251205291	68,02	1,23	++	
136	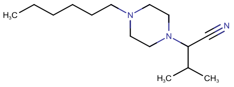	Z1252567819	67,56	0,83		
137	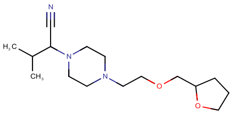	Z1256618686	67,83	1,10	++	
138	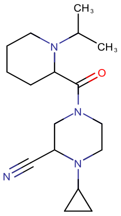	Z1257133062	67,47	0,74		
139	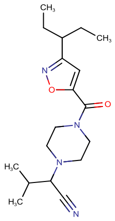	Z1261078980	68,26	1,55	++	YES
140	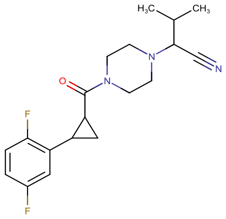	Z1261079001	67,81	1,10	++	

141	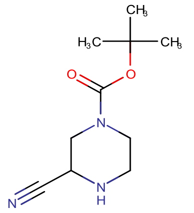	Z1267861824	67,72	1,01	++	
142	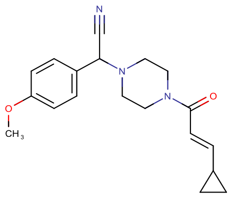	Z1273233592	68,26	1,55	++	
143	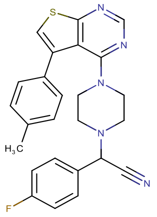	Z128166070	66,85	0,06		
144	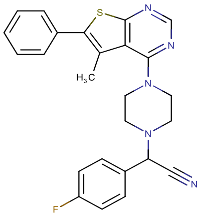	Z128166562	66,85	0,06		
145	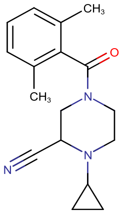	Z1296446860	67,18	0,47		
146	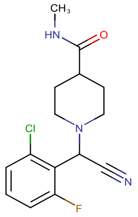	Z1297989190	68,26	1,55	++	YES
147	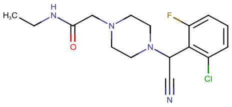	Z1298088664	67,90	1,19	++	

148	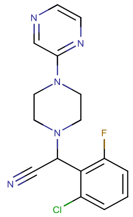	Z1298112865	67,36	0,65		
149	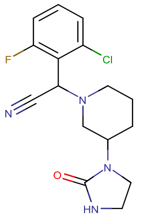	Z1298330150	68,26	1,55	++	YES
150	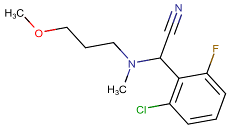	Z1298394455	67,83	1,10	++	
151	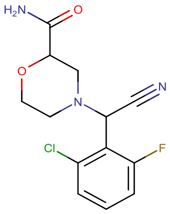	Z1298687439	66,82	0,11		
152	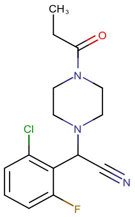	Z1298868401	67,20	0,47		
153	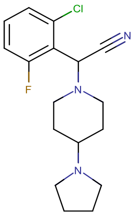	Z1298962751	67,54	0,83		
154	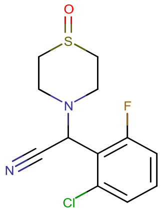	Z1298963715	66,82	0,11		

155	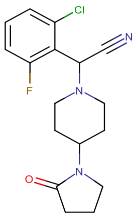	Z1298979831	68,35	1,64	++	YES
156	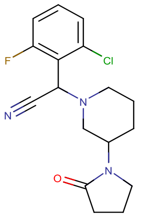	Z1299204981	67,99	1,28	++	
157	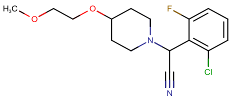	Z1299362572	68,01	1,28	++	
158	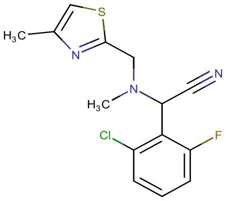	Z1299413124	67,27	0,56		
159	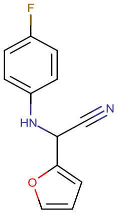	Z131022682	68,56	1,77	++	YES
160	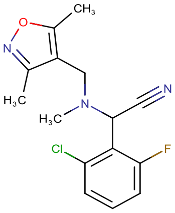	Z1310346224	67,90	1,19	++	
161	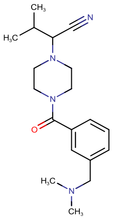	Z1314049925	67,29	0,56		

162	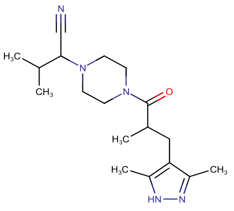	Z1314049962	67,83	1,10	++	
163	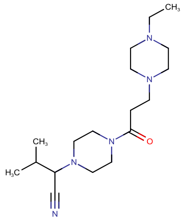	Z1314049972	67,18	0,47		
164	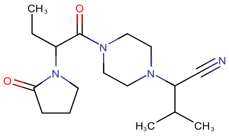	Z1314050030	67,90	1,19	++	
165	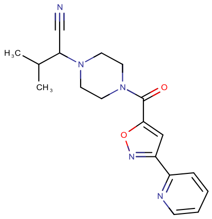	Z1314050038	68,08	1,37	++	
166	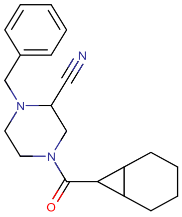	Z1315039908	67,54	0,83		
167	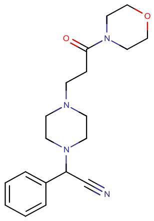	Z1317905230	67,74	1,01	++	
168	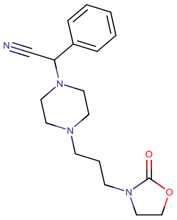	Z1317905252	68,17	1,46	++	

169	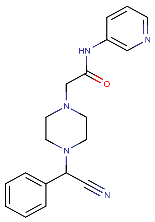	Z1317905264	68,01	1,28	++	
170	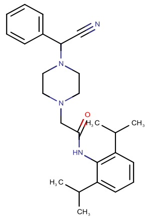	Z1317905289	67,90	1,19	++	
171	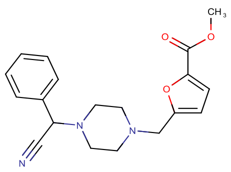	Z1317905301	67,92	1,19	++	
172	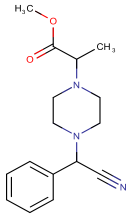	Z1317905425	67,83	1,10	++	
173	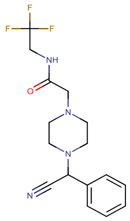	Z1317905463	68,37	1,63	++	YES
174	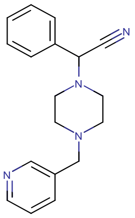	Z1317905741	68,26	1,55	++	YES
175	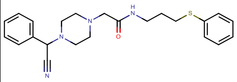	Z1317905871	68,08	1,37	++	

176	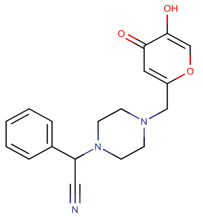	Z1317905971	67,99	1,28	++	
177	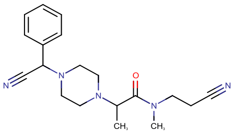	Z1317906051	67,92	1,19	++	
178	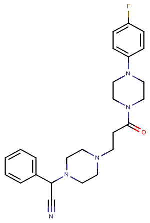	Z1317906092	67,72	1,01	++	
179	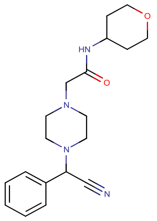	Z1317906129	68,17	1,46	++	YES
180	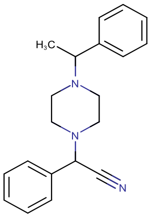	Z1317906144	67,63	0,92		
181	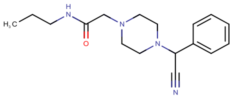	Z1317906181	67,83	1,10	++	
182	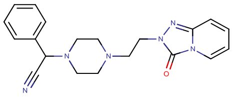	Z1317906286	68,08	1,37	++	

183	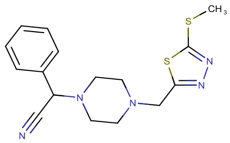	Z1317906315	68,08	1,37	++	
184	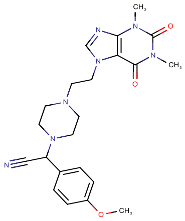	Z1317907646	68,26	1,55	++	
185	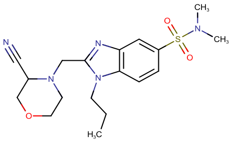	Z1318054522	66,82	0,11		
186	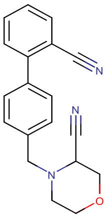	Z1318054576	67,11	0,38		
187	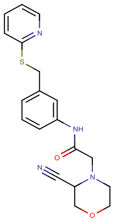	Z1318054622	66,91	0,20		
188	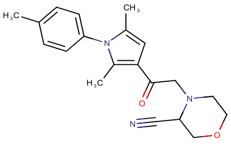	Z1318054744	66,91	0,20		
189	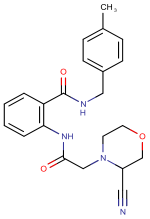	Z1318055496	66,73	0,02		

190	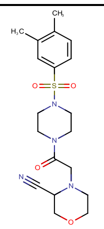	Z1318055731	67,09	0,38		
191	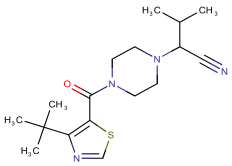	Z1322755479	68,26	1,55	++	YES
192	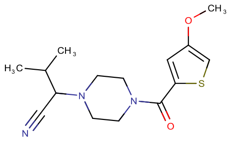	Z1322755486	68,01	1,28	++	
193	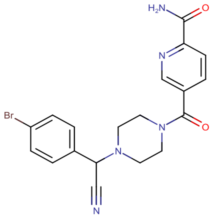	Z1323739354	67,27	0,56		
194	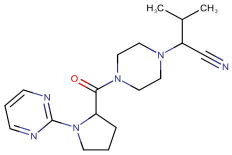	Z1325138958	67,90	1,19	++	
195	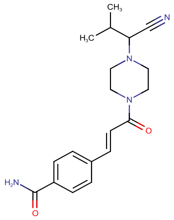	Z1325138998	67,83	1,10	++	
196	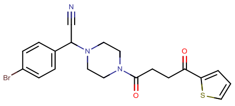	Z1332715801	67,36	0,65		

197	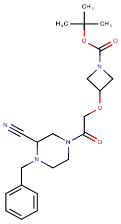	Z1332922453	67,36	0,65		
198	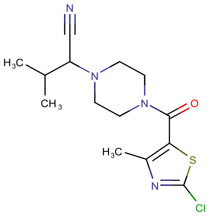	Z1335143805	67,83	1,10	++	
199	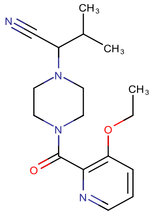	Z1336878609	67,45	0,74		
200	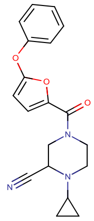	Z1336978286	67,47	0,74		
201	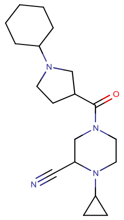	Z1338182566	67,29	0,56		
202	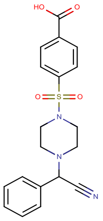	Z133943892	67,57	0,78		
203	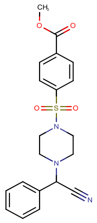	Z133944000	67,12	0,33		

204	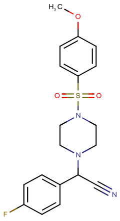	Z133944154	67,57	0,78		
205	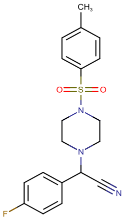	Z133944180	67,39	0,60		
206	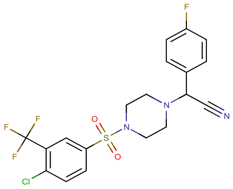	Z133944218	67,03	0,24		
207	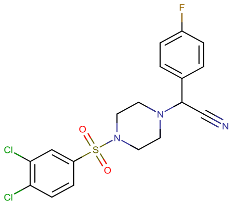	Z133944228	66,94	0,15		
208	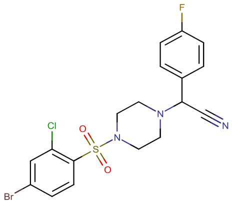	Z133944366	66,94	0,15		
209	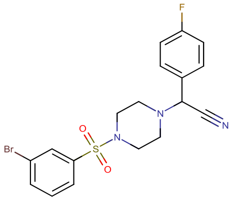	Z133944404	67,21	0,42		
210	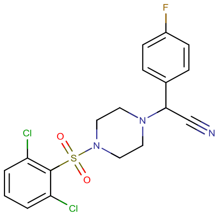	Z133944418	67,03	0,24		

211	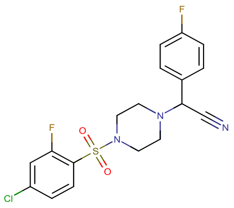	Z133944468	67,21	0,42		
212	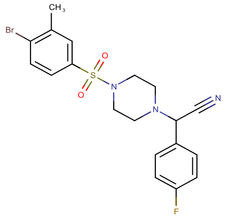	Z133944520	67,12	0,33		
213	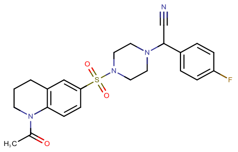	Z133944754	67,66	0,87		
214	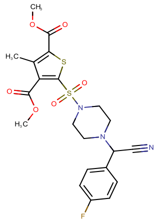	Z133944792	67,03	0,24		
215	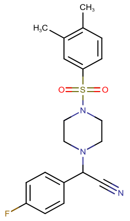	Z133944856	67,30	0,51		
216	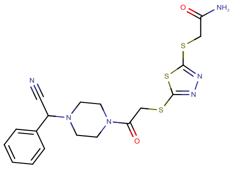	Z1341534692	67,36	0,65		
217	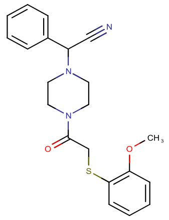	Z1341536129	67,27	0,56		

218	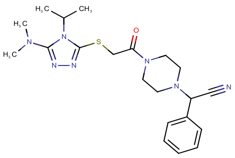	Z1341536452	67,36	0,65		
219	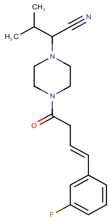	Z1343956502	68,17	1,46	++	YES
220	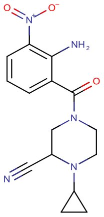	Z1344097840	67,27	0,56		
221	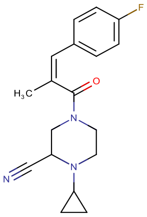	Z1344097879	67,09	0,38		
222	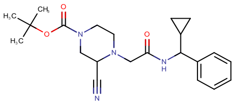	Z1346454174	67,45	0,74		
223	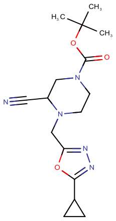	Z1346454408	66,93	0,20		
224	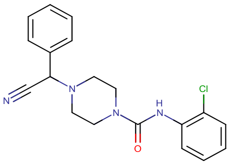	Z135326554	67,18	0,47		

225	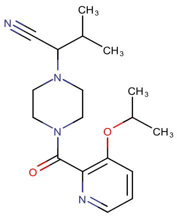	Z1354925755	67,74	1,01	++	
226	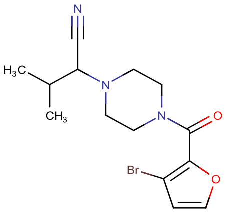	Z1360343319	67,74	1,01	++	
227	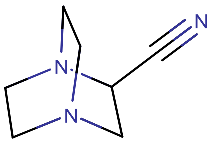	Z1362381716	66,46	-0,25		
228	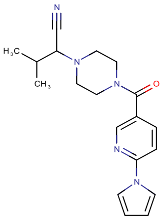	Z1374064216	67,72	1,01	++	
229	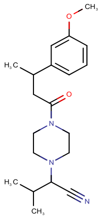	Z1374064231	68,01	1,28	++	
230	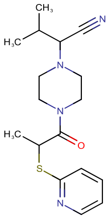	Z1379178933	67,63	0,92		
231	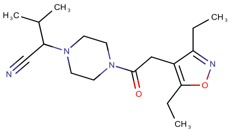	Z1390085782	68,26	1,55	++	YES

232	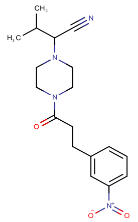	Z1390085789	67,90	1,19	++	
233	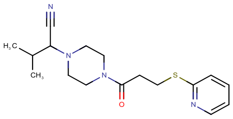	Z1390085811	67,38	0,65		
234	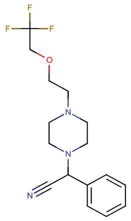	Z1395303116	68,10	1,37	++	
235	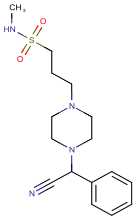	Z1395303258	68,44	1,73	++	YES
236	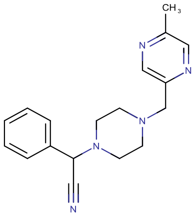	Z1395303263	67,99	1,28	++	
237	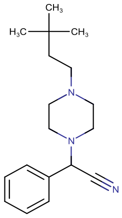	Z1395303287	67,63	0,92		
238	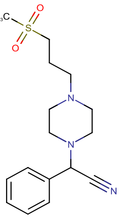	Z1395303380	67,27	0,56		

239	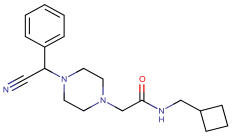	Z1395304494	68,44	1,73	++	YES
240	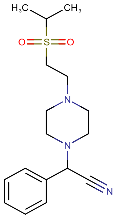	Z1395304566	68,10	1,37	++	
241	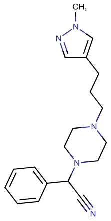	Z1395305786	67,99	1,28	++	
242	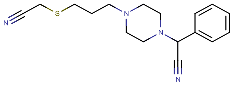	Z1395305911	68,26	1,55	++	
243	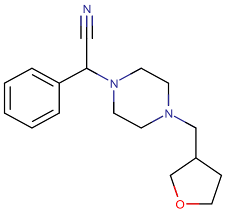	Z1395305944	68,17	1,46	++	YES
244	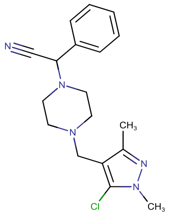	Z1395306416	67,92	1,19	++	
245	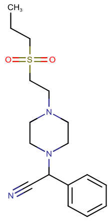	Z1395306999	67,81	1,10	++	

246	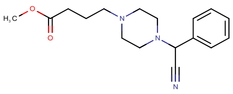	Z1395307034	67,90	1,19	++	
247	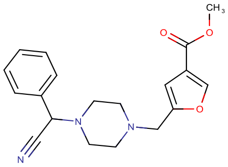	Z1395307112	67,81	1,10	++	
248	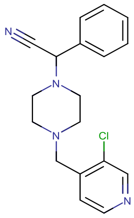	Z1395307186	67,90	1,19	++	
249	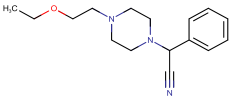	Z1395307265	67,90	1,19	++	
250	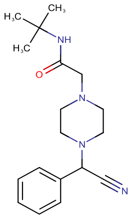	Z1395308621	68,17	1,46	++	
251	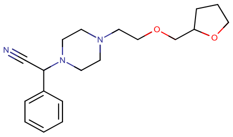	Z1395309709	67,29	0,56		
252	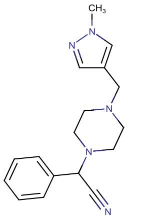	Z1395310822	67,81	1,10	++	

253	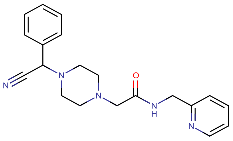	Z1395310861	67,29	0,56		
254	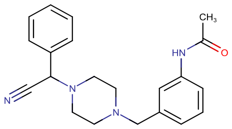	Z1395310863	67,83	1,10	++	
255	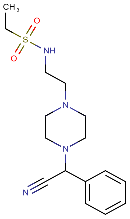	Z1395310896	68,17	1,46	++	
256	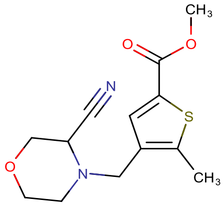	Z1400644030	66,84	0,11		
257	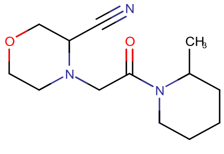	Z1400644248	67,11	0,38		
258	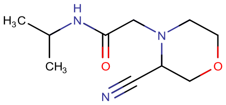	Z1400651783	67,00	0,29		
259	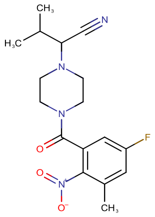	Z1411162206	68,08	1,37	++	

260	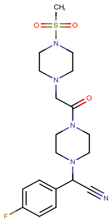	Z1416461382	67,54	0,83		
261	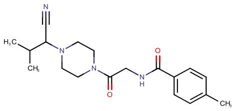	Z1416783341	68,44	1,73	++	YES
262	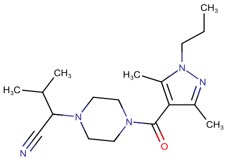	Z1416783344	67,92	1,19	++	
263	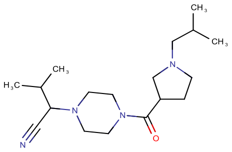	Z1419905587	68,01	1,28	++	
264	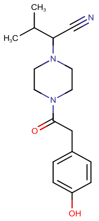	Z1426561309	67,63	0,92		
265	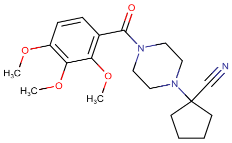	Z146371830	67,75	0,96		
266	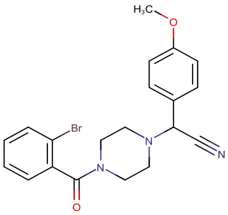	Z146384194	67,81	1,10	++	

267	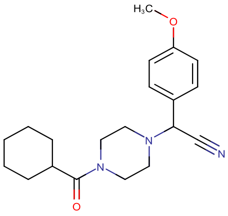	Z146384300	68,35	1,64	++	YES
268	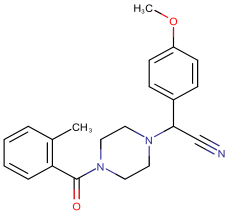	Z146384516	67,81	1,10	++	
269	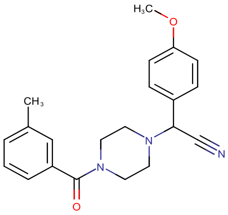	Z146384632	68,11	1,32	++	
270	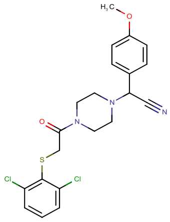	Z146386830	67,75	0,96		
271	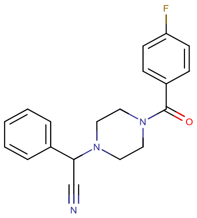	Z146786584	67,66	0,87		
272	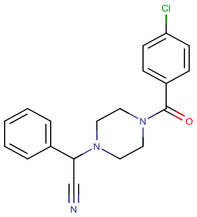	Z146786590	67,48	0,69		
273	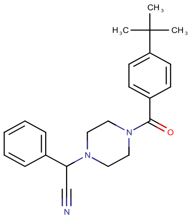	Z146786610	67,84	1,05	++	

274	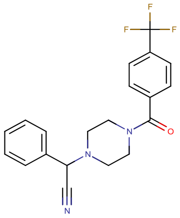	Z146786620	67,66	0,87		
275	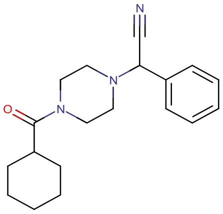	Z146786666	68,02	1,23	++	
276	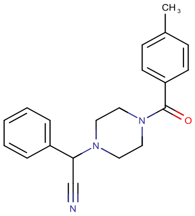	Z146786702	67,66	0,87		
277	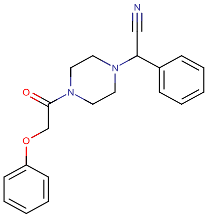	Z146786704	67,54	0,83		
278	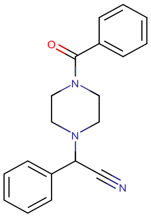	Z146786712	68,29	1,50	++	YES
279	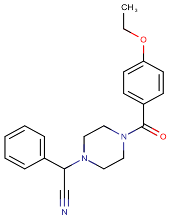	Z146786810	67,57	0,78		
280	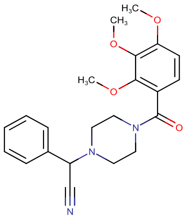	Z146786812	67,57	0,78		

281	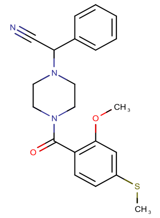	Z146786856	67,36	0,65		
282	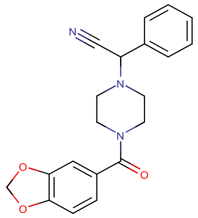	Z146786864	67,93	1,14	++	
283	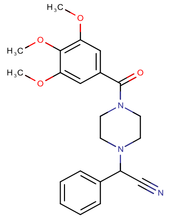	Z146786866	67,66	0,87		
284	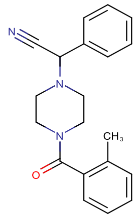	Z146786882	67,48	0,69		
285	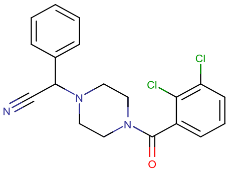	Z146786896	67,21	0,42		
286	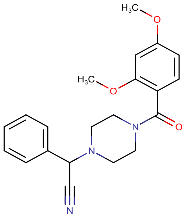	Z146786908	67,54	0,83		
287	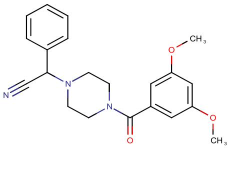	Z146786910	67,84	1,05	++	

288	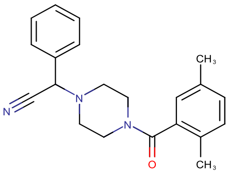	Z146786916	67,75	0,96		
289	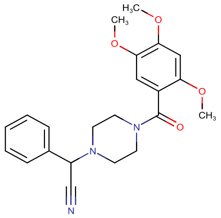	Z146786936	67,75	0,96		
290	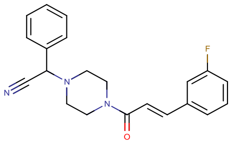	Z146786952	67,36	0,65		
291	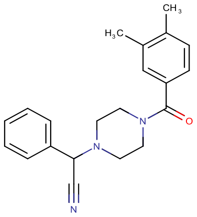	Z146787000	67,84	1,05	++	
292	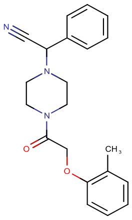	Z146787066	67,66	0,87		
293	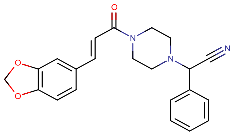	Z146787084	67,84	1,05	++	
294	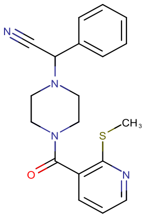	Z146787160	67,57	0,78		

295	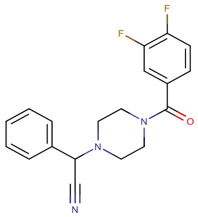	Z146787218	67,48	0,69		
296	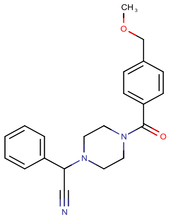	Z146788104	67,48	0,69		
297	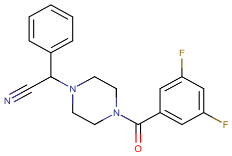	Z146788700	67,39	0,60		
298	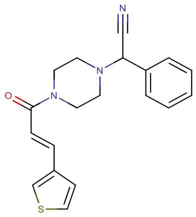	Z146789554	67,93	1,14	++	
299	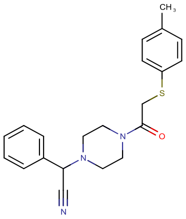	Z146789566	67,66	0,87		
300	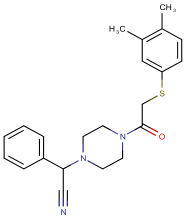	Z146789568	67,57	0,78		
301	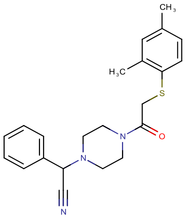	Z146789570	67,39	0,60		

302	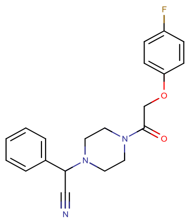	Z146790068	67,75	0,96		
303	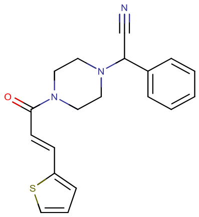	Z146790748	67,66	0,87		
304	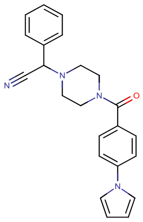	Z146791184	67,48	0,69		
305	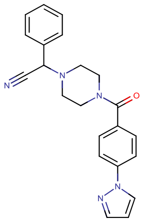	Z146791650	67,57	0,78		
306	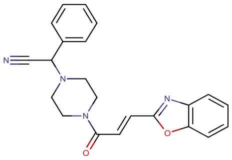	Z146791804	67,75	0,96		
307	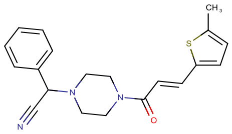	Z146792448	67,84	1,05	++	
308	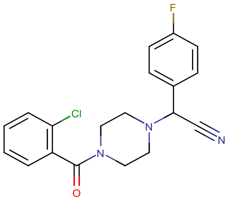	Z146792834	67,48	0,69		

309	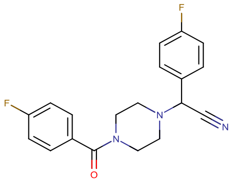	Z146792856	67,48	0,69		
310	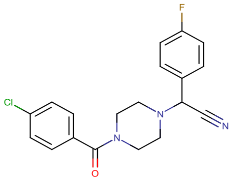	Z146792862	67,66	0,87		
311	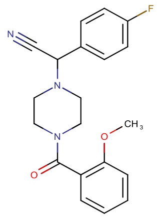	Z146792874	67,66	0,87		
312	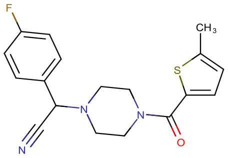	Z146792888	67,66	0,87		
313	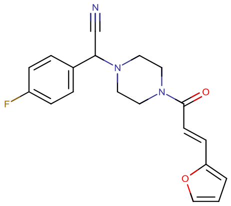	Z146792898	67,66	0,87		
314	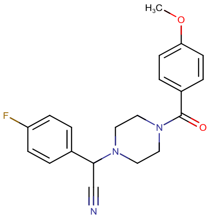	Z146792908	67,75	0,96		
315	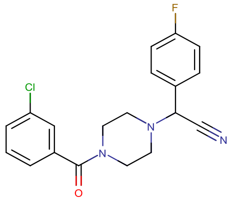	Z146792920	67,75	0,96		

316	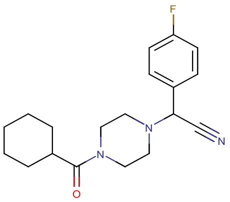	Z146792938	67,93	1,14	++	
317	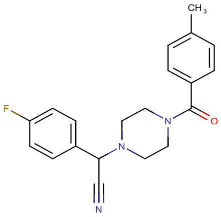	Z146792974	67,66	0,87		
318	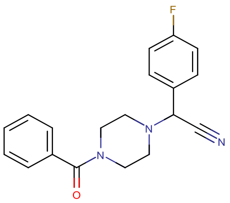	Z146792984	67,84	1,05	++	
319	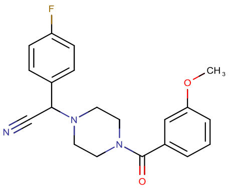	Z146793000	67,48	0,69		
320	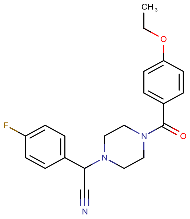	Z146793082	67,57	0,78		
321	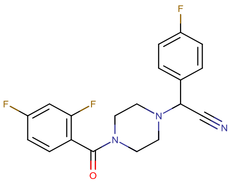	Z146793104	67,75	0,96		
322	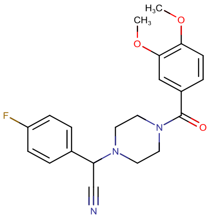	Z146793120	67,57	0,78		

323	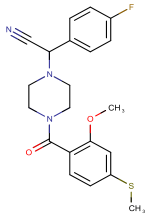	Z146793128	67,66	0,87		
324	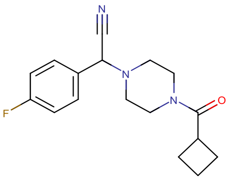	Z146793148	67,99	1,28	++	
325	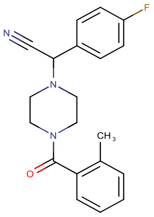	Z146793154	67,57	0,78		
326	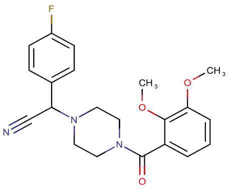	Z146793178	67,66	0,87		
327	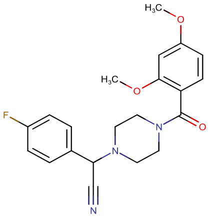	Z146793180	67,75	0,96		
328	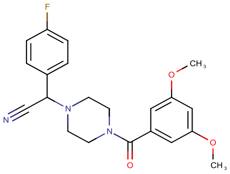	Z146793182	67,39	0,60		
329	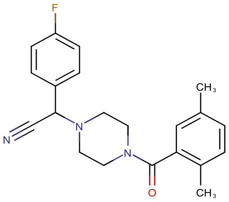	Z146793188	67,57	0,78		

330	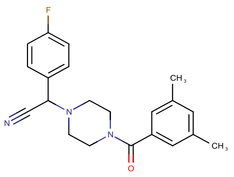	Z146793190	67,66	0,87		
331	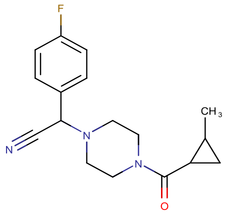	Z146793216	68,17	1,46	++	
332	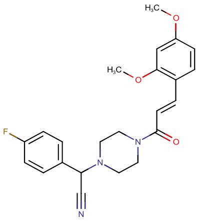	Z146793234	67,84	1,05	++	
333	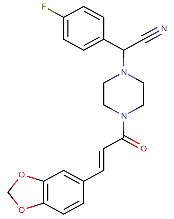	Z146793354	67,75	0,96		
334	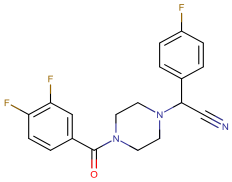	Z146793486	67,66	0,87		
335	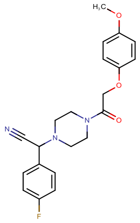	Z146793866	67,66	0,87		
336	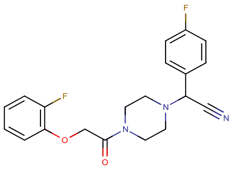	Z146794016	67,75	0,96		

337	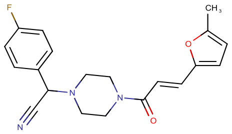	Z146794314	67,30	0,51		
338	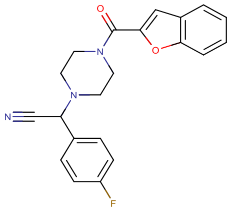	Z146794366	67,66	0,87		
339	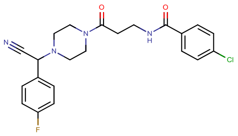	Z146794676	67,66	0,87		
340	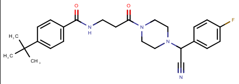	Z146794680	67,84	1,05	++	
341	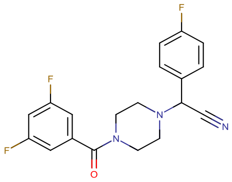	Z146794930	67,66	0,87		
342	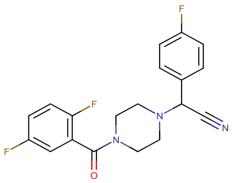	Z146795282	67,66	0,87		
343	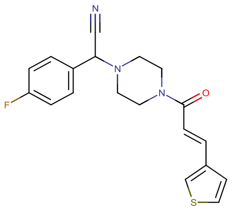	Z146795774	67,66	0,87		

344	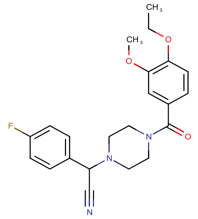	Z146796090	67,48	0,69		
345	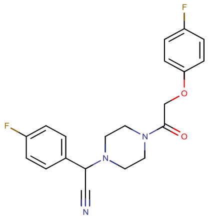	Z146796282	67,48	0,69		
346	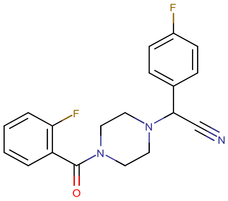	Z146796530	67,57	0,78		
347	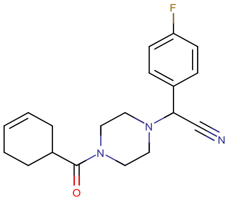	Z146796862	67,84	1,05	++	
348	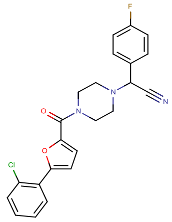	Z146796890	66,85	0,06		
349	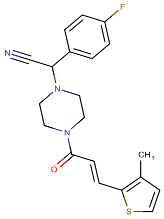	Z146796900	67,03	0,24		
350	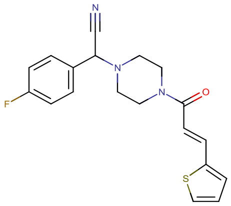	Z146796956	67,75	0,96		

351	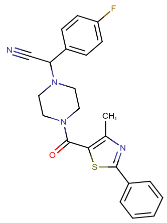	Z146797074	67,39	0,60		
352	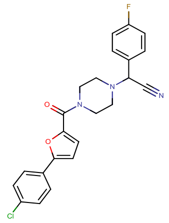	Z146797206	67,12	0,33		
353	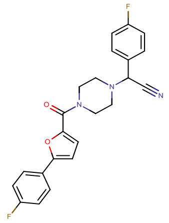	Z146797222	67,39	0,60		
354	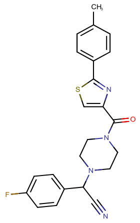	Z146797390	67,30	0,51		
355	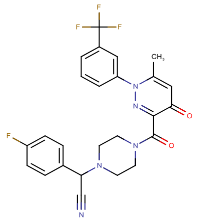	Z146797486	67,84	1,05	++	
356	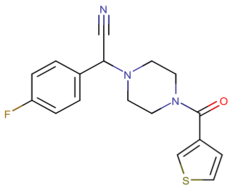	Z146797682	67,57	0,78		
357	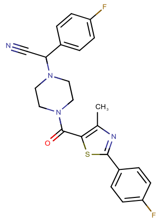	Z146797698	67,39	0,60		

358	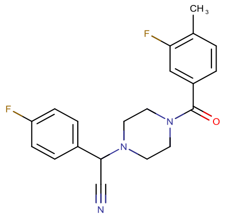	Z146797770	67,75	0,96		
359	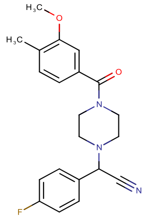	Z146797878	67,75	0,96		
360	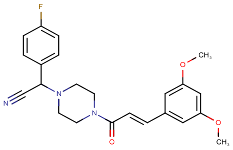	Z146798042	67,03	0,24		
361	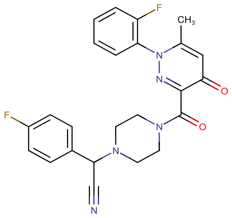	Z146798134	67,48	0,69		
362	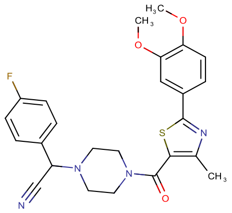	Z146798180	67,57	0,78		
363	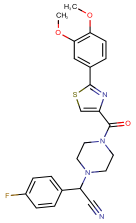	Z146798318	67,48	0,69		
364	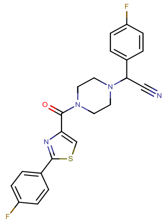	Z146798334	67,48	0,69		

365	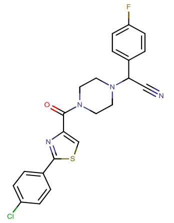	Z146798344	67,30	0,51		
366	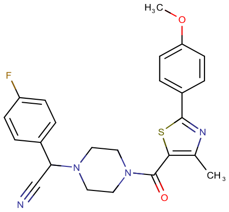	Z146798360	67,30	0,51		
367	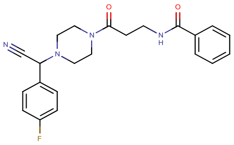	Z146798442	67,75	0,96		
368	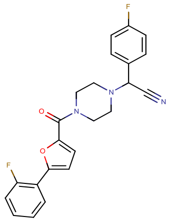	Z146798586	67,12	0,33		
369	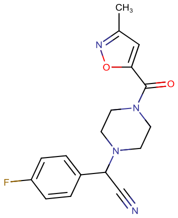	Z146798600	67,48	0,69		
370	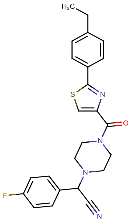	Z146798682	67,03	0,24		
371	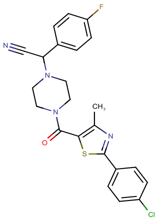	Z146798990	67,21	0,42		

372	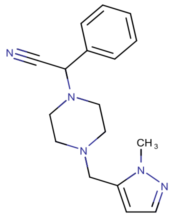	Z1470770969	67,81	1,10	++	
373	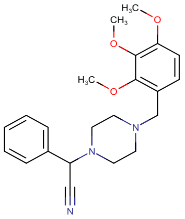	Z1470771103	67,72	1,01	++	
374	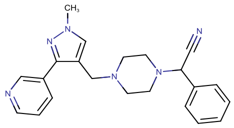	Z1470771430	68,08	1,37	++	YES
375	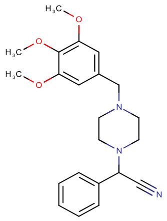	Z1470771926	67,90	1,19	++	
376	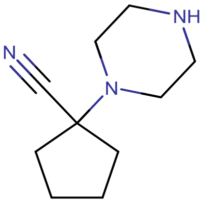	Z147652266	67,57	0,78		
377	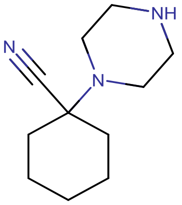	Z147652268	67,84	1,05	++	
378	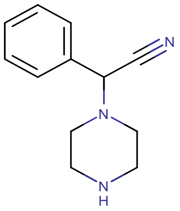	Z147652270	67,48	0,69		

379	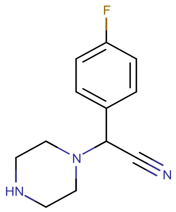	Z147652272	67,39	0,60		
380	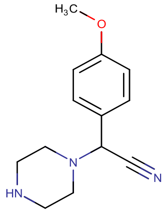	Z147652274	67,75	0,96		
381	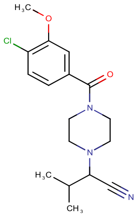	Z1527939173	68,08	1,37	++	
382	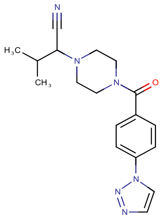	Z1536253826	67,99	1,28	++	
383	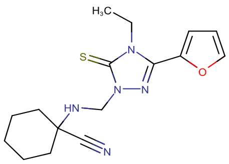	Z1544844693	67,99	1,28	++	
384	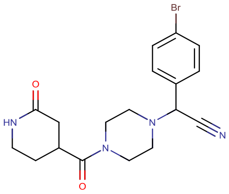	Z1559418017	67,54	0,83		
385	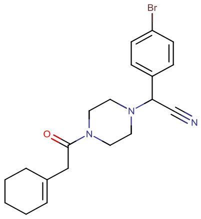	Z1559424893	67,09	0,38		

386	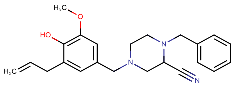	Z1561891976	67,18	0,47		
387	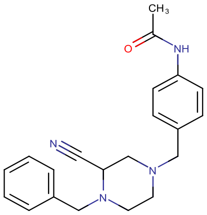	Z1561892062	67,18	0,47		
388	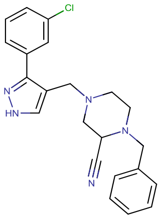	Z1561892438	67,27	0,56		
389	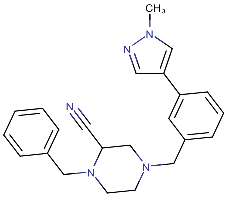	Z1561892657	66,64	-0,07		
390	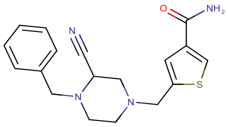	Z1561892800	67,48	0,69		
391	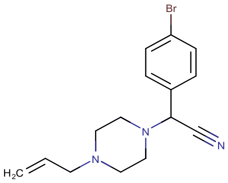	Z1567199572	67,45	0,74		
392	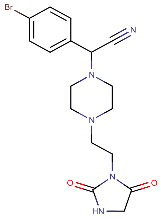	Z1567199718	67,99	1,28	++	YES

393	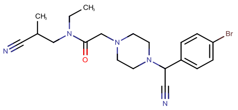	Z1567199760	67,90	1,19	++	
394	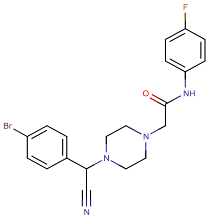	Z1567200756	67,90	1,19	++	
395	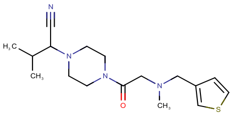	Z1567275046	67,90	1,19	++	
396	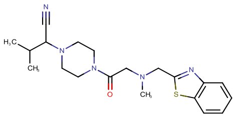	Z1567275135	68,17	1,46	++	
397	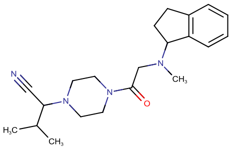	Z1567276344	66,84	0,11		
398	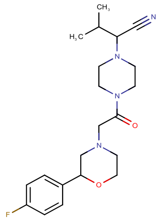	Z1567277098	68,26	1,55	++	YES
399	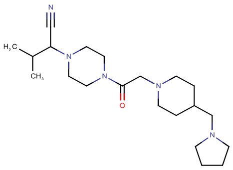	Z1567278739	67,72	1,01	++	

400	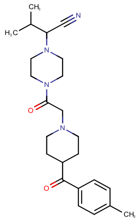	Z1567280757	68,17	1,46	++	YES
401	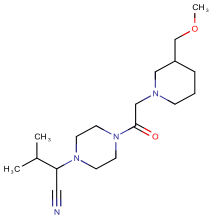	Z1567287508	67,38	0,65		
402	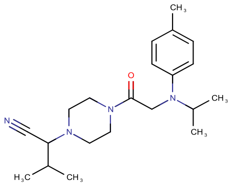	Z1567293695	68,44	1,73	++	YES
403	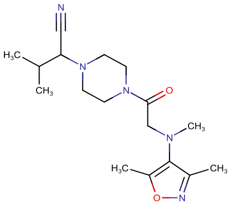	Z1567295437	67,90	1,19	++	
404	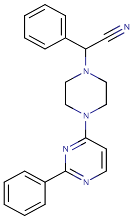	Z1567825382	67,18	0,47		
405	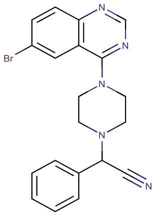	Z1567825392	67,18	0,47		
406	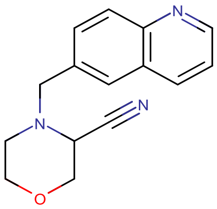	Z1569087060	66,93	0,20		

407	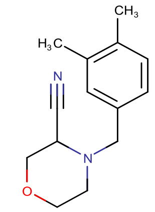	Z1569087407	67,27	0,56		
408	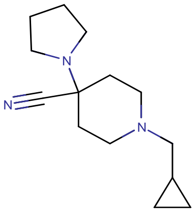	Z1587277493	67,27	0,56		
409	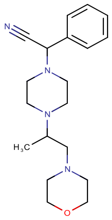	Z1587287536	67,74	1,01	++	
410	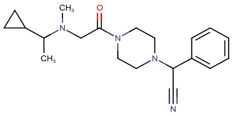	Z1587360325	67,27	0,56		
411	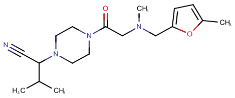	Z1587361452	67,74	1,01	++	
412	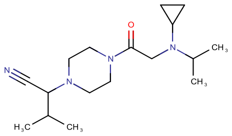	Z1587376071	68,10	1,37	++	
413	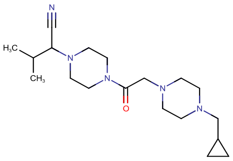	Z1587379787	67,83	1,10	++	

414	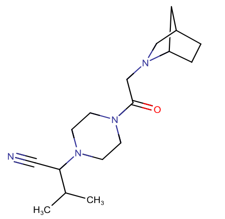	Z1587387563	67,83	1,10	++	
415	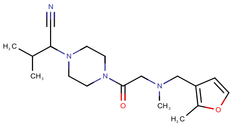	Z1587389446	68,08	1,37	++	
416	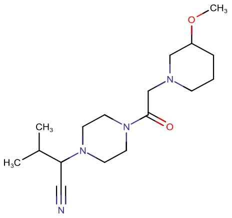	Z1587437781	68,01	1,28	++	
417	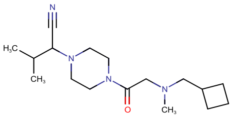	Z1587486553	68,19	1,45	++	
418	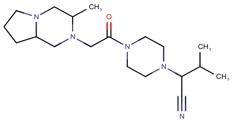	Z1587494645	68,08	1,37	++	
419	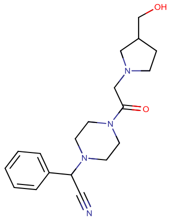	Z1587513554	67,36	0,65		
420	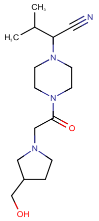	Z1587513580	67,56	0,83		

421	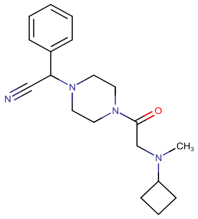	Z1587521645	66,66	-0,07		
422	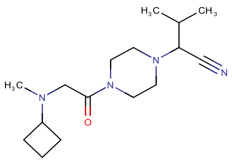	Z1587521664	68,10	1,37	++	
423	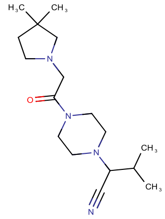	Z1587532393	68,26	1,55	++	
424	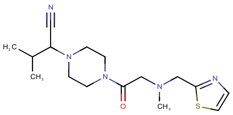	Z1587537151	67,47	0,74		
425	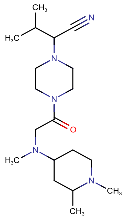	Z1587541978	67,99	1,28	++	
426	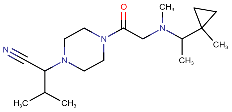	Z1587550804	67,90	1,19	++	
427	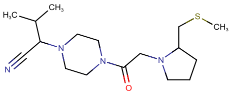	Z1587567923	67,83	1,10	++	

428	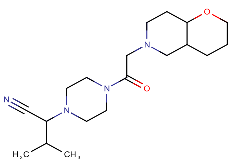	Z1587569267	68,01	1,28	++	
429	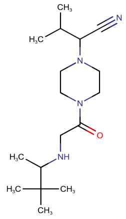	Z1587641595	68,26	1,55	++	
430	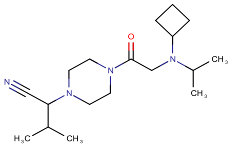	Z1587648894	67,29	0,56		
431	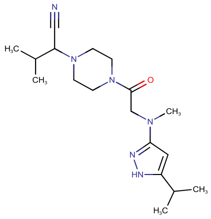	Z1587649769	68,26	1,55	++	
432	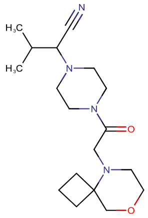	Z1587705252	68,08	1,37	++	
433	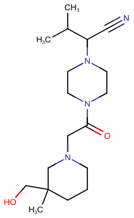	Z1587710951	67,65	0,92		
434	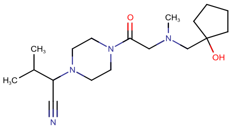	Z1587711050	68,26	1,55	++	

435	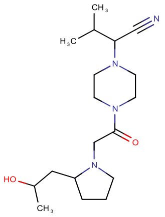	Z1587711149	68,01	1,28	++	
436	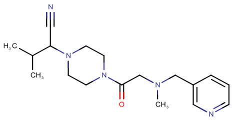	Z1587714592	68,19	1,45	++	
437	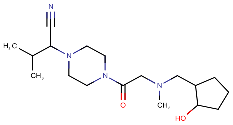	Z1587731409	68,08	1,37	++	
438	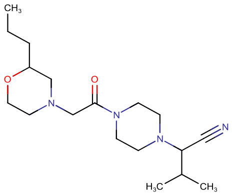	Z1587737878	67,92	1,19	++	
439	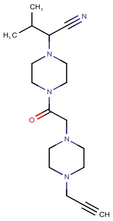	Z1587748705	68,44	1,73	++	
440	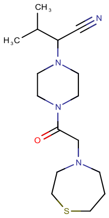	Z1587767868	68,53	1,82	++	
441	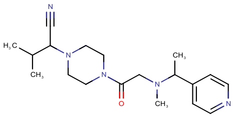	Z1587767943	68,17	1,46	++	

442	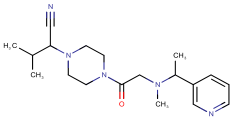	Z1587768000	67,56	0,83		
443	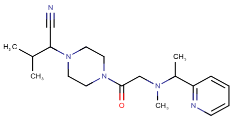	Z1587768057	67,81	1,10	++	
444	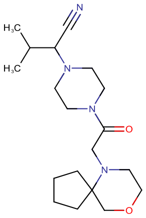	Z1587864600	68,35	1,64	++	
445	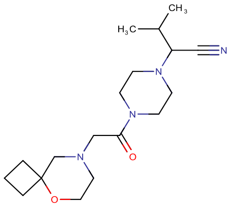	Z1587867496	68,08	1,37	++	
446	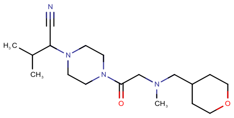	Z1587895251	67,56	0,83		
447	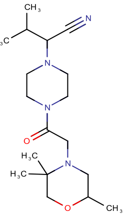	Z1587906431	68,26	1,55	++	
448	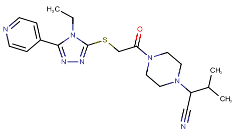	Z1588933085	68,08	1,37	++	

449	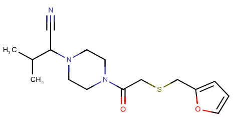	Z1588935685	67,63	0,92		
450	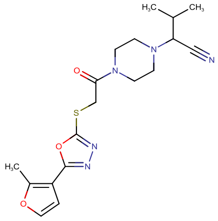	Z1588941429	68,17	1,46	++	
451	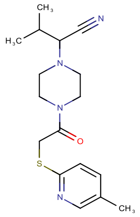	Z1588952211	68,17	1,46	++	
452	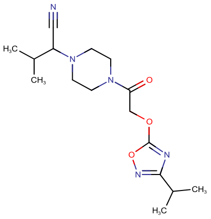	Z1588955305	68,08	1,37	++	
453	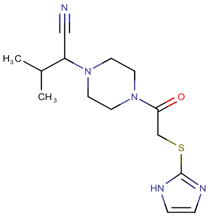	Z1588956093	68,26	1,55	++	
454	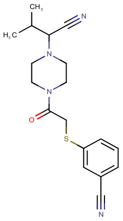	Z1588956911	68,08	1,37	++	
455	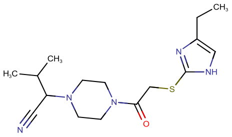	Z1588957279	68,17	1,46	++	

456	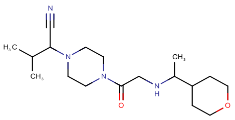	Z1590441584	68,01	1,28	++	
457	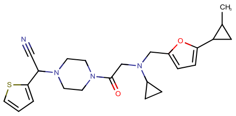	Z1592182133	67,63	0,92		
458	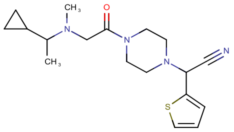	Z1592184195	67,90	1,19	++	
459	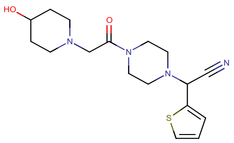	Z1592185225	67,90	1,19	++	
460	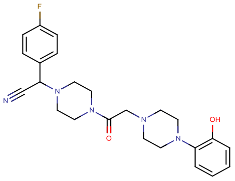	Z1592185527	67,72	1,01	++	
461	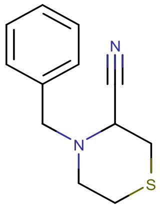	Z1592571492	67,27	0,56		
462	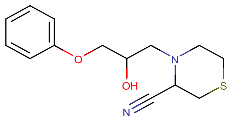	Z1592571539	67,65	0,92		

463	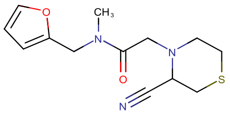	Z1592571810	67,02	0,29		
464	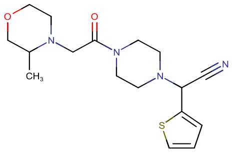	Z1592572831	67,99	1,28	++	
465	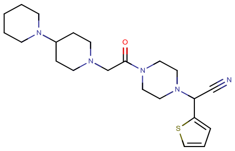	Z1592573255	67,90	1,19	++	
466	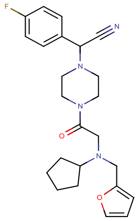	Z1592573313	67,36	0,65		
467	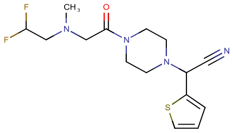	Z1594992780	67,99	1,28	++	
468	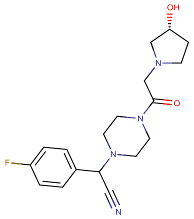	Z1594994021	67,36	0,65		
469	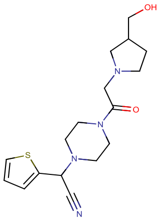	Z1595067527	67,81	1,10	++	

470	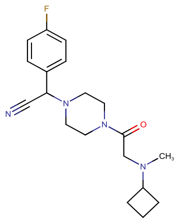	Z1595075447	67,63	0,92		
471	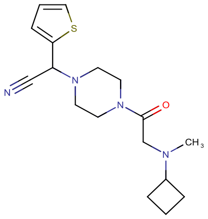	Z1595075506	67,74	1,01	++	
472	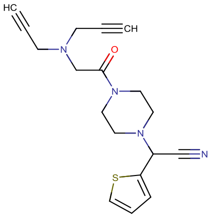	Z1595091474	67,99	1,28	++	
473	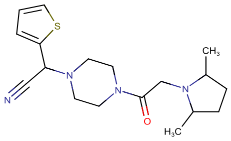	Z1595113295	67,90	1,19	++	
474	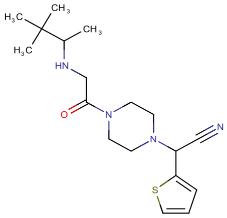	Z1595195668	67,83	1,10	++	
475	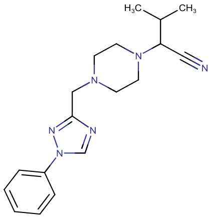	Z1595320918	68,17	1,46	++	
476	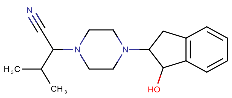	Z1595325025	67,81	1,10	++	

477	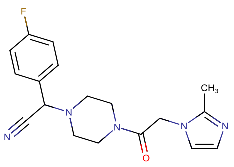	Z1607441302	67,72	1,01	++	
478	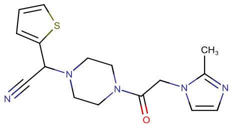	Z1613059249	67,99	1,28	++	
479	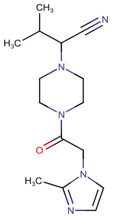	Z1613065957	67,99	1,28	++	
480	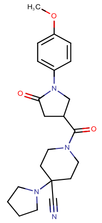	Z1613899622	67,09	0,38		
481	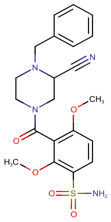	Z1614698022	67,81	1,10	++	
482	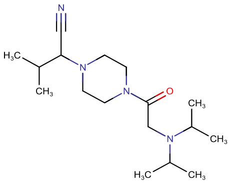	Z1619962558	67,65	0,92		
483	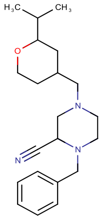	Z1620133576	67,11	0,38		

484	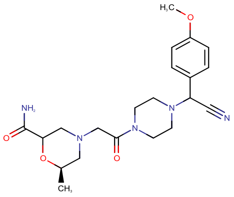	Z1621798136	67,45	0,74		
485	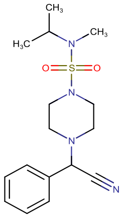	Z1626212253	67,56	0,83		
486	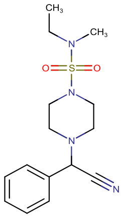	Z1626212461	66,66	-0,07		
487	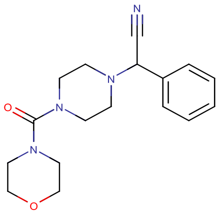	Z1626212814	67,90	1,19	++	
488	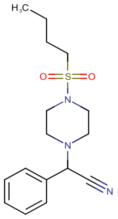	Z1626212827	67,36	0,65		
489	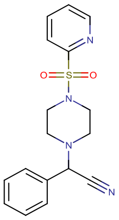	Z1626213046	67,18	0,47		
490	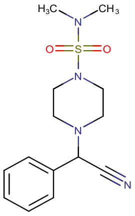	Z1626213199	67,29	0,56		

491	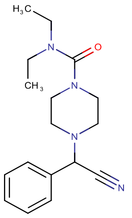	Z1626213397	67,83	1,10	++	
492	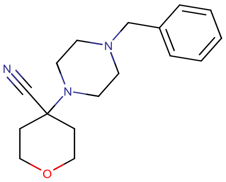	Z1627006367	67,99	1,28	++	
493	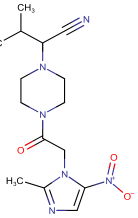	Z1627140074	67,90	1,19	++	
494	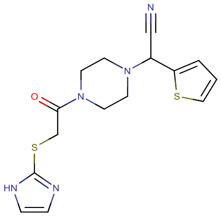	Z1627150502	68,08	1,37	++	
495	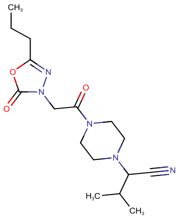	Z1627154598	68,17	1,46	++	
496	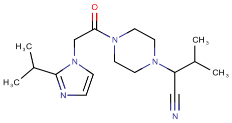	Z1627171307	68,26	1,55	++	
497	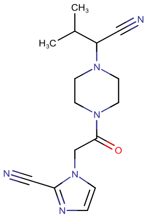	Z1627185535	67,92	1,19	++	

498	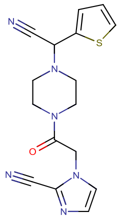	Z1627185580	68,08	1,37	++	
499	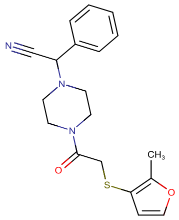	Z1631872491	66,93	0,20		
500	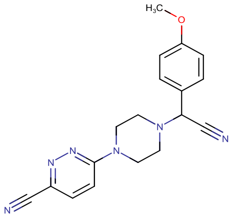	Z1632954774	67,72	1,01	++	
501	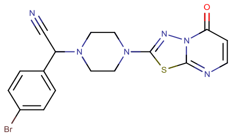	Z1632955862	67,09	0,38		
502	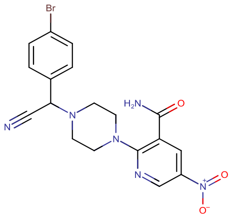	Z1632955990	66,64	-0,07		
503	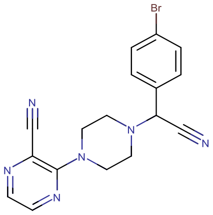	Z1632956001	67,27	0,56		
504	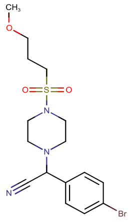	Z1633136614	67,09	0,38		

505	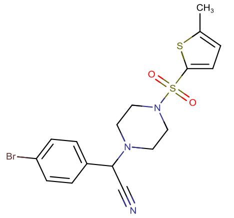	Z1633136681	66,46	-0,25		
506	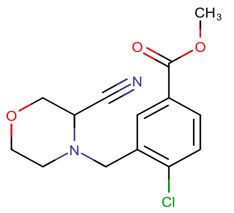	Z1636004939	67,09	0,38		
507	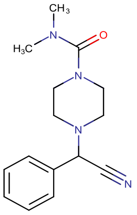	Z1643042925	68,26	1,55	++	
508	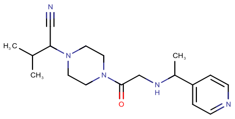	Z1646488085	67,83	1,10	++	
509	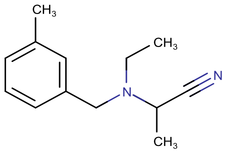	Z1651955041	66,66	-0,07		
510	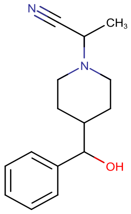	Z1651972818	67,20	0,47		
511	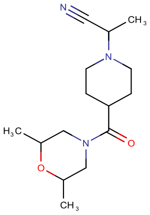	Z1651994022	67,99	1,28	++	

512	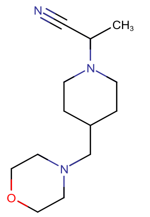	Z1652001211	67,92	1,19	++	
513	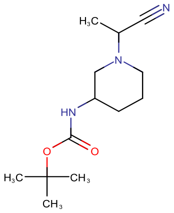	Z1652017626	68,08	1,37	++	
514	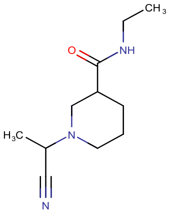	Z1652060484	68,35	1,64	++	
515	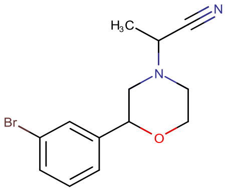	Z1652064128	67,83	1,10	++	
516	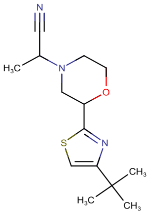	Z1652066584	67,92	1,19	++	
517	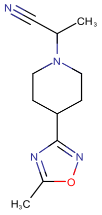	Z1652067084	68,08	1,37	++	
518	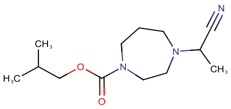	Z1652076350	67,11	0,38		

519	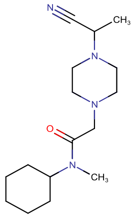	Z1652095858	67,65	0,92		
520	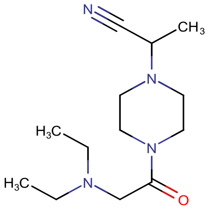	Z1652098084	67,47	0,74		
521	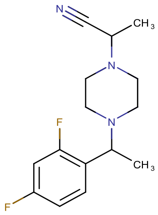	Z1652098759	67,92	1,19	++	
522	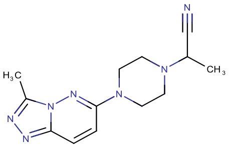	Z1652110801	67,92	1,19	++	
523	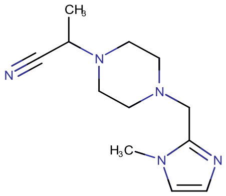	Z1652143530	68,08	1,37	++	
524	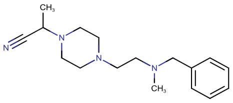	Z1652158431	67,83	1,10	++	
525	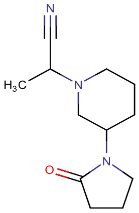	Z1652168247	67,20	0,47		

526	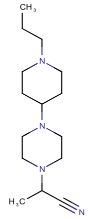	Z1652173028	67,74	1,01	++	
527	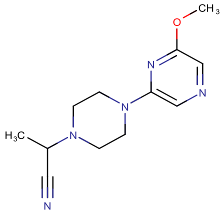	Z1652184157	68,44	1,73	++	
528	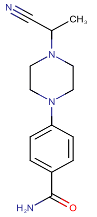	Z1652190474	68,17	1,46	++	
529	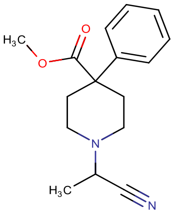	Z1652191429	68,08	1,37	++	
530	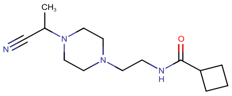	Z1652195288	67,99	1,28	++	
531	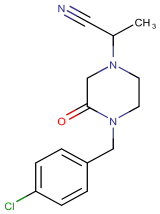	Z1652197096	67,99	1,28	++	
532	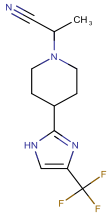	Z1652203467	67,99	1,28	++	

533	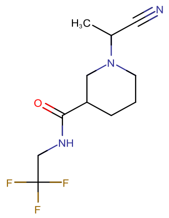	Z1652211940	67,63	0,92		
534	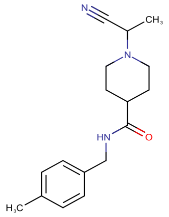	Z1652229659	67,90	1,19	++	
535	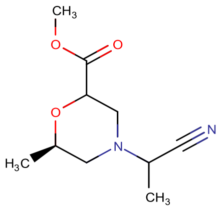	Z1652259145	68,01	1,28	++	
536	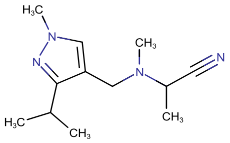	Z1652264880	67,02	0,29		
537	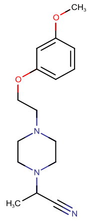	Z1652269184	67,92	1,19	++	
538	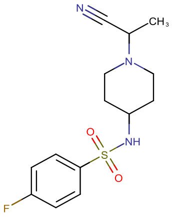	Z1652294950	67,81	1,10	++	
539	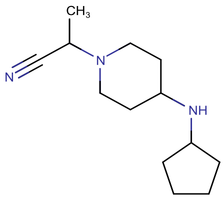	Z1652375975	67,65	0,92		

540	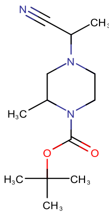	Z1652392406	67,47	0,74		
541	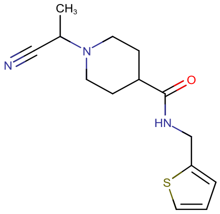	Z1652394347	67,72	1,01	++	
542	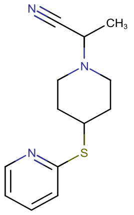	Z1652399616	68,08	1,37	++	
543	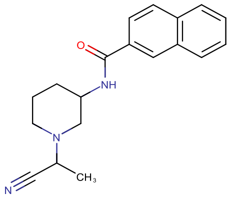	Z1652409471	67,72	1,01	++	
544	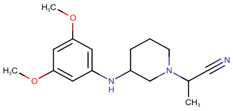	Z1652414003	68,26	1,55	++	
545	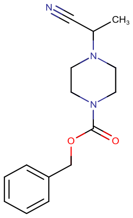	Z1652435905	68,01	1,28	++	
546	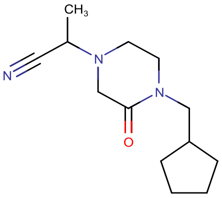	Z1652448453	68,17	1,46	++	

547	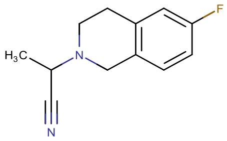	Z1652521774	67,38	0,65		
548	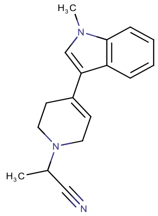	Z1652595241	67,72	1,01	++	
549	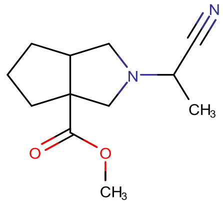	Z1652682440	67,83	1,10	++	
550	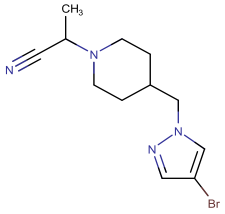	Z1652684936	67,65	0,92		
551	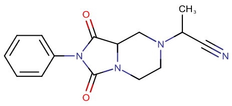	Z1652685944	68,17	1,46	++	
552	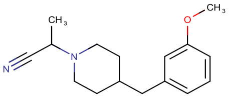	Z1652705006	67,65	0,92		
553	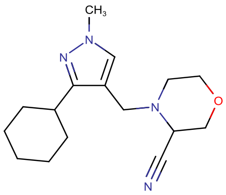	Z1661233918	66,84	0,11		

554	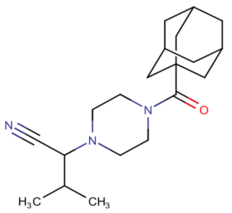	Z166623370	68,38	1,59	++	
555	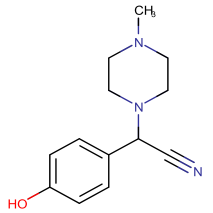	Z166632344	68,29	1,50	++	
556	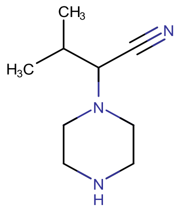	Z168879940	67,75	0,96		
557	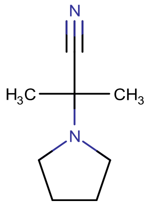	Z168893480	67,47	0,74		
558	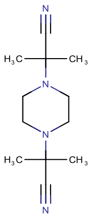	Z168911126	68,47	1,68	++	
559	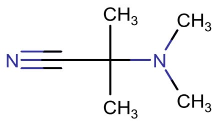	Z168912518	66,85	0,06		
560	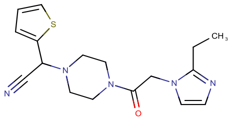	Z1692658205	67,81	1,10	++	

561	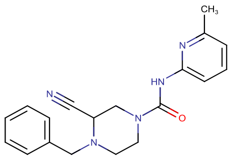	Z1693397020	66,55	-0,16		
562	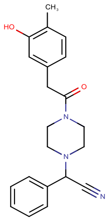	Z1694712621	67,45	0,74		
563	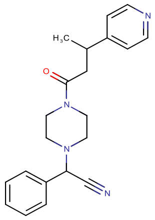	Z1694712709	67,45	0,74		
564	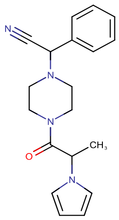	Z1694713172	67,54	0,83		
565	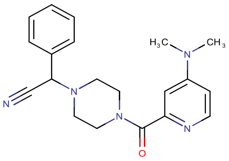	Z1694714031	67,81	1,10	++	
566	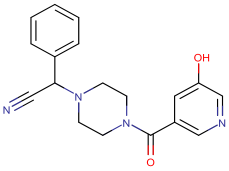	Z1694714510	67,54	0,83		
567	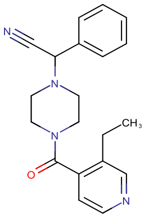	Z1694714957	67,36	0,65		

568	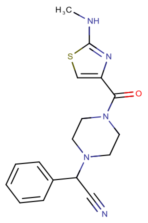	Z1694714985	67,63	0,92		
569	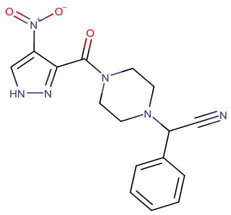	Z1694716606	67,27	0,56		
570	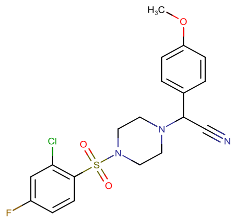	Z1695670036	67,45	0,74		
571	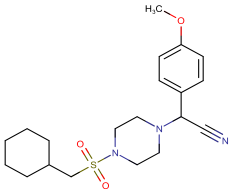	Z1695670186	67,36	0,65		
572	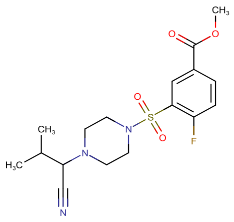	Z1695672916	67,81	1,10	++	
573	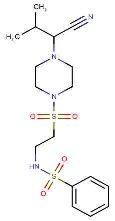	Z1695673081	68,17	1,46	++	
574	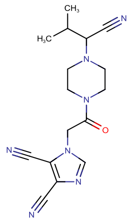	Z1696937367	68,17	1,46	++	

575	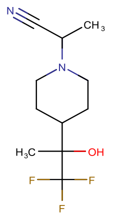	Z1700671585	67,47	0,74		
576	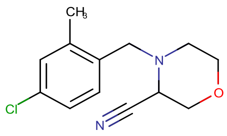	Z1702973584	66,93	0,20		
577	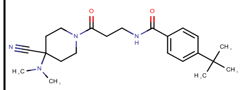	Z1725828924	67,36	0,65		
578	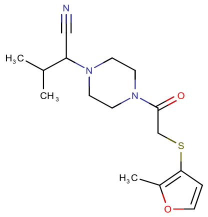	Z1729810580	67,74	1,01	++	
579	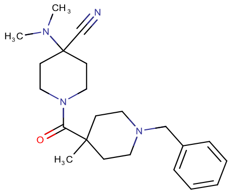	Z1736233013	66,55	-0,16		
580	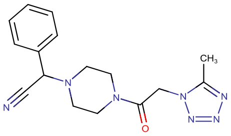	Z1742706129	67,45	0,74		
581	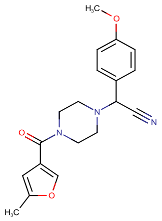	Z1763590888	68,08	1,37	++	

582	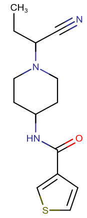	Z1769989984	67,81	1,10	++	
583	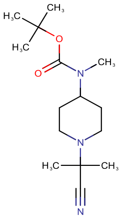	Z1770101086	67,00	0,29		
584	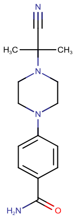	Z1770126428	67,99	1,28	++	
585	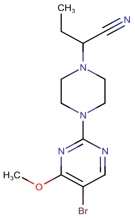	Z1770164963	67,45	0,74		
586	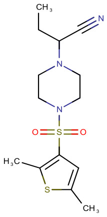	Z1770199280	67,99	1,28	++	
587	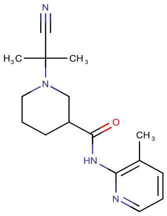	Z1770242452	67,02	0,29		
588	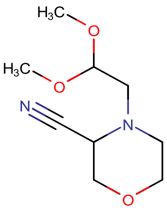	Z1770246083	67,02	0,29		

589	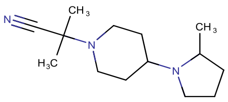	Z1770263317	66,84	0,11		
590	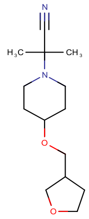	Z1770284792	67,11	0,38		
591	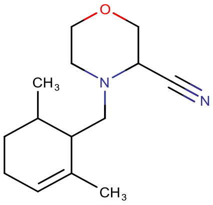	Z1799134223	67,20	0,47		
592	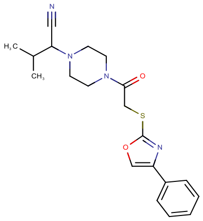	Z1883026071	68,17	1,46	++	
593	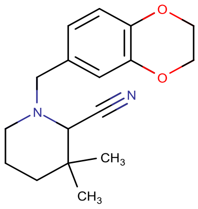	Z1887781406	66,84	0,11		
594	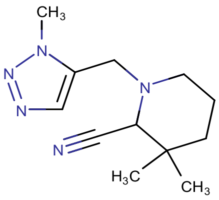	Z1898070917	66,46	-0,25		
595	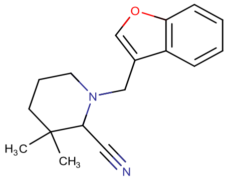	Z1898071202	66,55	-0,16		

596	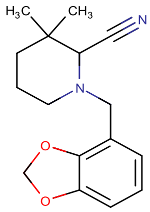	Z1898071531	66,64	-0,07		
597	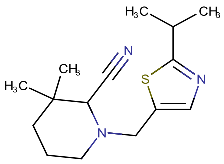	Z1898072431	66,57	-0,16		
598	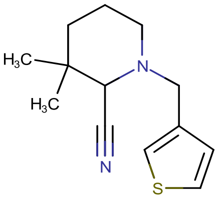	Z1898072589	66,93	0,20		
599	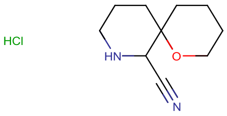	Z1910147414	67,00	0,29		
600	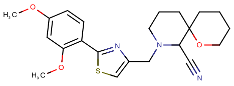	Z1930494492	66,46	-0,25		
601	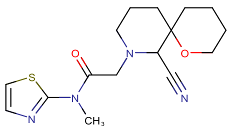	Z1930500746	66,64	-0,07		
602	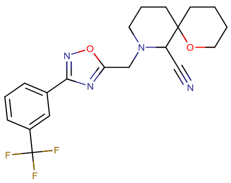	Z1930502686	66,46	-0,25		

603	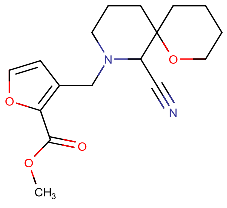	Z1930502982	66,73	0,02		
604	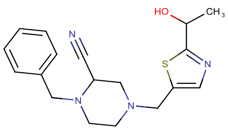	Z1935317732	67,18	0,47		
605	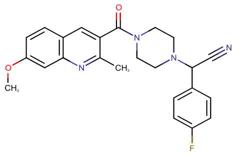	Z195230414	67,21	0,42		
606	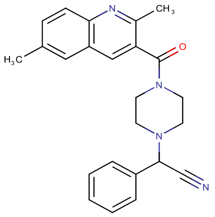	Z197906024	67,75	0,96		
607	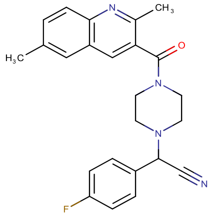	Z197906130	67,57	0,78		
608	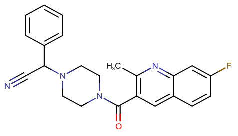	Z199891266	67,39	0,60		
609	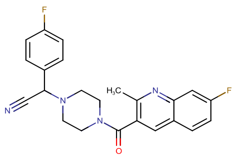	Z199891474	67,75	0,96		

610	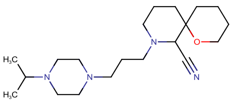	Z2002380915	66,73	0,02		
611	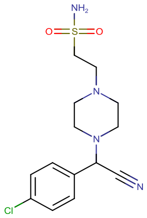	Z2016067157	68,53	1,82	++	
612	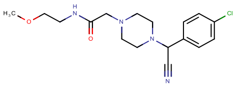	Z2016069212	67,90	1,19	++	
613	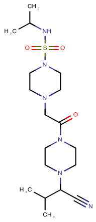	Z2036960757	68,08	1,37	++	
614	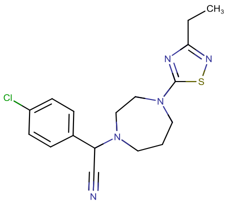	Z2037031956	68,26	1,55	++	
615	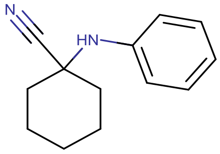	Z204018286	68,38	1,59	++	
616	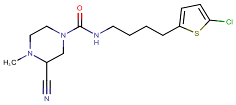	Z2044059784	68,08	1,37	++	

617	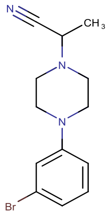	Z2045825356	68,44	1,73	++	
618	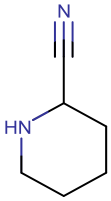	Z205012332	66,75	0,02		
619	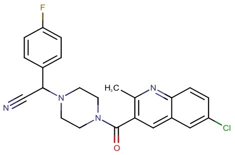	Z217448698	67,57	0,78		
620	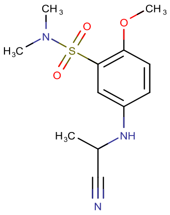	Z219839452	67,48	0,69		
621	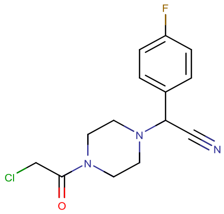	Z220323530	67,48	0,69		
622	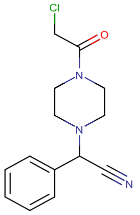	Z220323534	67,39	0,60		
623	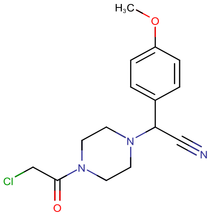	Z220323536	68,29	1,50	++	

624	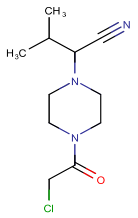	Z220346776	68,56	1,77	++	
625	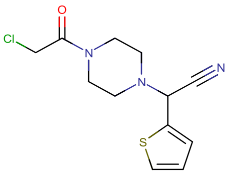	Z220346778	68,20	1,41	++	
626	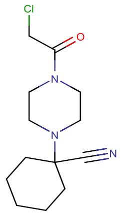	Z220346780	67,12	0,33		
627	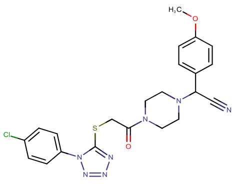	Z220380744	67,48	0,69		
628	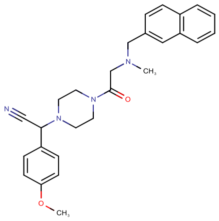	Z220382222	67,75	0,96		
629	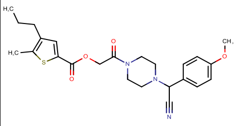	Z220389008	67,57	0,78		
630	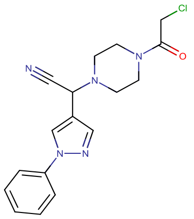	Z220442154	68,11	1,32	++	

631	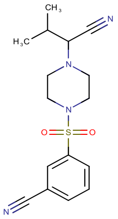	Z223333868	68,17	1,46	++	
632	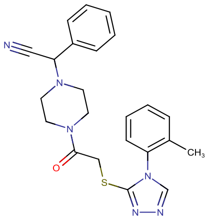	Z223793260	67,72	1,01	++	
633	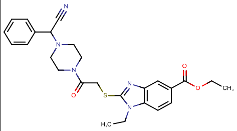	Z223800396	67,30	0,51		
634	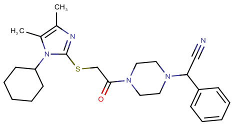	Z223810008	67,27	0,56		
635	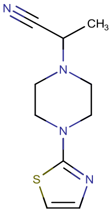	Z224842582	68,56	1,77	++	
636	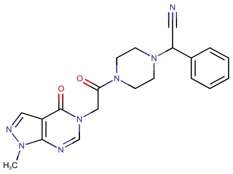	Z230755634	67,54	0,83		
637	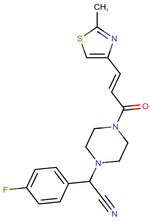	Z230756548	67,84	1,05	++	

638	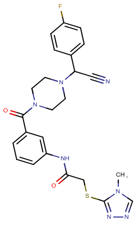	Z230756882	68,02	1,23	++	
639	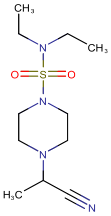	Z231707810	68,38	1,59	++	
640	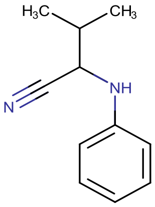	Z237487004	67,21	0,42		
641	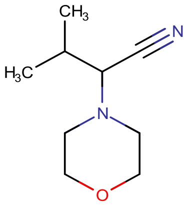	Z237515164	68,47	1,68	++	
642	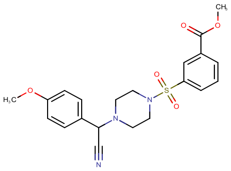	Z240253470	67,54	0,83		
643	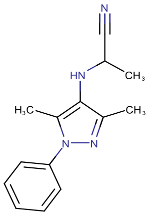	Z240957088	67,02	0,29		
644	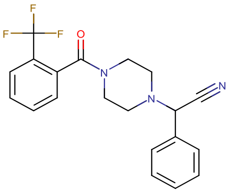	Z245157224	67,75	0,96		

645	HCl 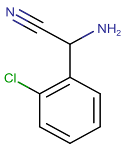	Z247612592	67,48	0,69		
646	HCl 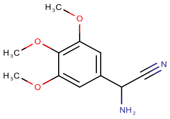	Z247612594	67,48	0,69		
647	HCl 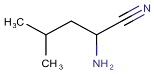	Z247612598	68,02	1,23	++	
648	HCl 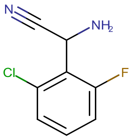	Z247612602	67,03	0,24		
649	HCl 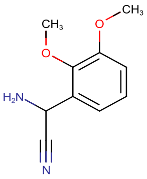	Z247612604	68,65	1,86	++	
650	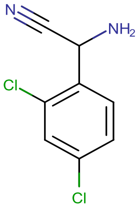	Z247612606	67,66	0,87		
651	HCl 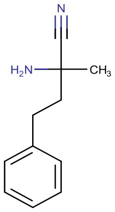	Z247612608	67,93	1,14	++	

652	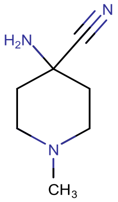	Z247612626	67,56	0,83		
653	HCl 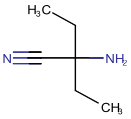	Z247612656	68,56	1,77	++	
654	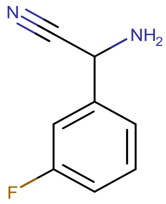	Z247612662	66,76	-0,03		
655	HCl 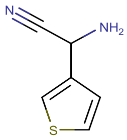	Z247612676	68,47	1,68	++	
656	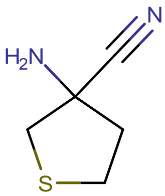	Z247612682	66,94	0,15		
657	HCl 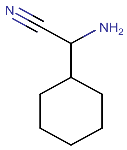	Z247612718	67,45	0,74		
658	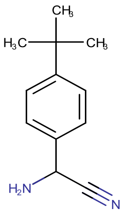	Z247612722	67,90	1,19	++	

659	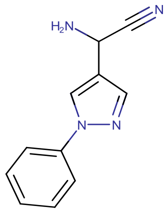	Z247612804	67,39	0,60		
660	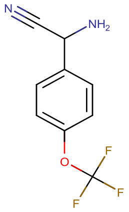	Z247612834	66,85	0,06		
661	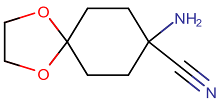	Z247612838	69,01	2,22	++	
662	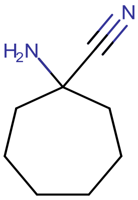	Z247612850	67,38	0,65		
663	HCl 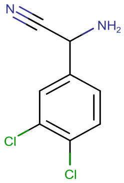	Z247612860	67,57	0,78		
664	HCl 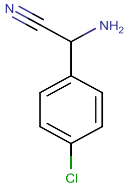	Z247612866	67,39	0,60		
665	HCl 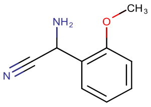	Z247612884	68,47	1,68	++	

666	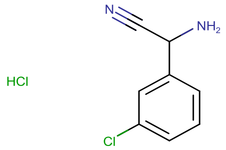	Z247612886	67,75	0,96		
667	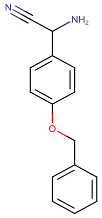	Z247612892	68,20	1,41	++	
668	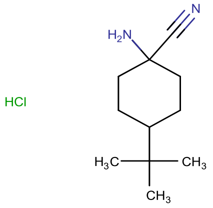	Z247612898	68,56	1,77	++	
669	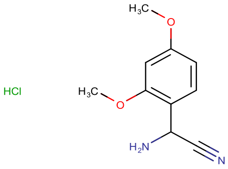	Z247612930	68,65	1,86	++	
670	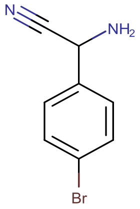	Z247612940	66,94	0,15		
671	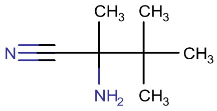	Z247612948	66,73	0,02		
672	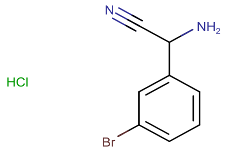	Z247612958	67,18	0,47		

673	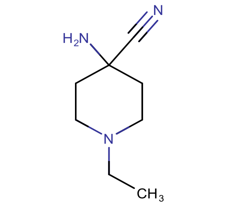	Z247612968	67,90	1,19	++	
674	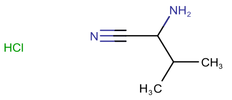	Z247612982	67,12	0,33		
675	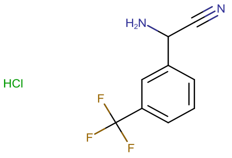	Z247612992	67,39	0,60		
676	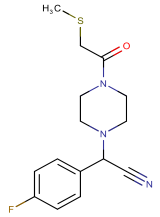	Z254072016	67,66	0,87		
677	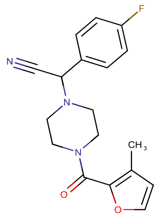	Z254073388	67,39	0,60		
678	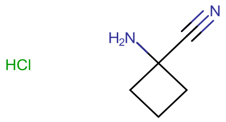	Z255102782	67,03	0,24		
679	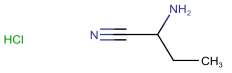	Z270840828	68,02	1,23	++	

680	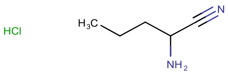	Z270840838	68,02	1,23	++	
681	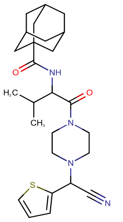	Z283656280	68,11	1,32	++	
682	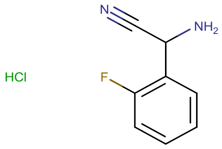	Z283861232	68,11	1,32	++	
683	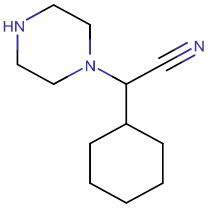	Z285165208	68,92	2,13	++	
684	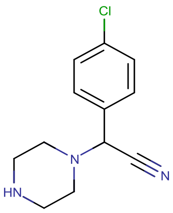	Z285165356	67,45	0,74		
685	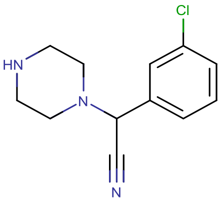	Z285165376	67,20	0,47		
686	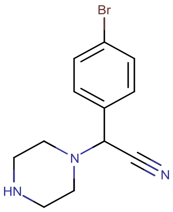	Z285165430	67,75	0,96		

687	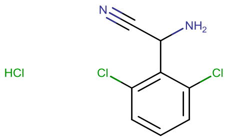	Z285165754	67,03	0,24		
688	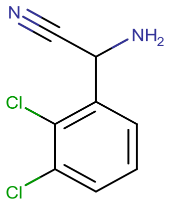	Z295458276	67,12	0,33		
689	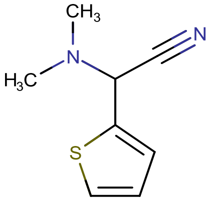	Z317025100	67,65	0,92		
690	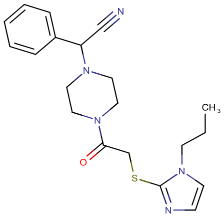	Z324654214	66,94	0,15		
691	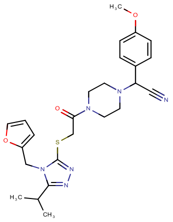	Z324663918	67,90	1,19	++	
692	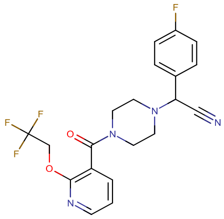	Z339982152	67,48	0,69		
693	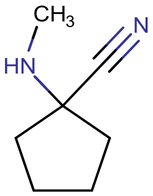	Z359508294	67,38	0,65		

694	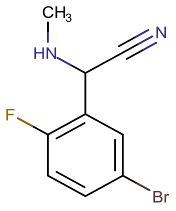	Z359508396	66,66	-0,07		
695	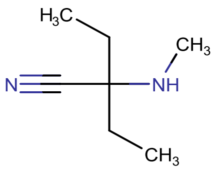	Z359508484	67,20	0,47		
696	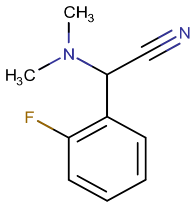	Z362638290	68,01	1,28	++	
697	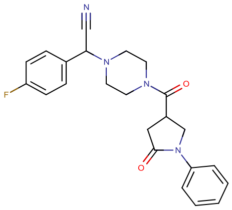	Z365557954	68,02	1,23	++	
698	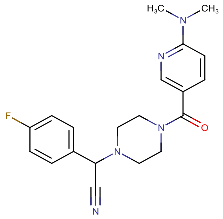	Z365558010	67,75	0,96		
699	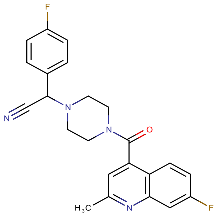	Z365558074	67,66	0,87		
700	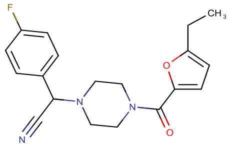	Z368219342	67,66	0,87		

701	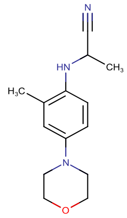	Z369454250	67,12	0,33		
702	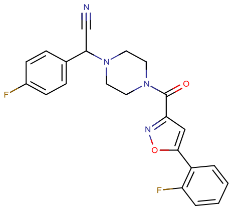	Z373674204	67,39	0,60		
703	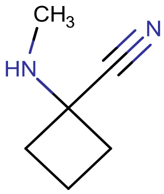	Z400377166	67,47	0,74		
704	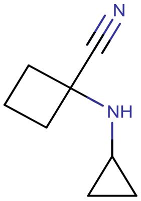	Z400377196	66,64	-0,07		
705	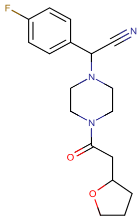	Z423417100	67,38	0,65		
706	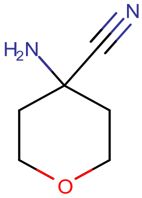	Z440604874	68,62	1,91	++	
707	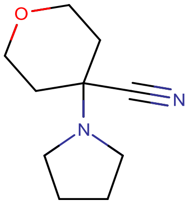	Z447995202	67,45	0,74		

708	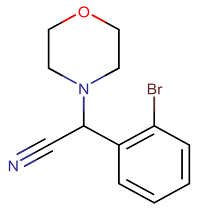	Z447996056	68,29	1,50	++	
709	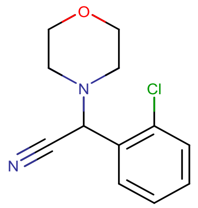	Z447996246	67,72	1,01	++	
710	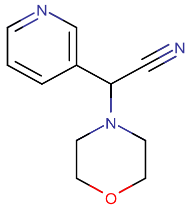	Z447996270	67,36	0,65		
711	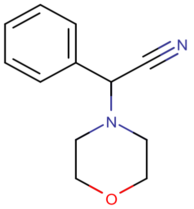	Z447996374	67,63	0,92		
712	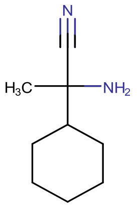	Z448247474	67,83	1,10	++	
713	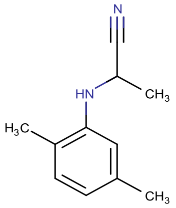	Z45201270	67,39	0,60		
714	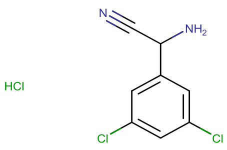	Z452435538	67,30	0,51		

715	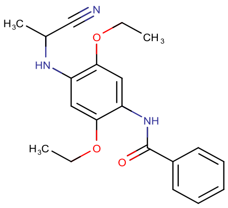	Z45260135	66,94	0,15		
716	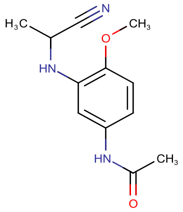	Z45261486	67,21	0,42		
717	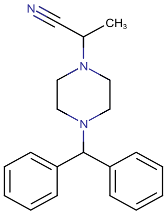	Z46169658	68,56	1,77	++	
718	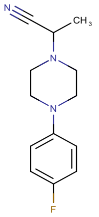	Z46196904	68,65	1,86	++	
719	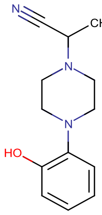	Z46200760	68,65	1,86	++	
720	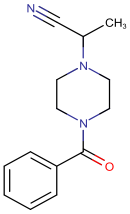	Z46403962	68,65	1,86	++	
721	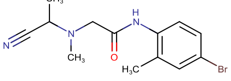	Z46435681	68,47	1,68	++	

722	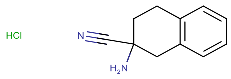	Z489295784	68,35	1,64	++	
723	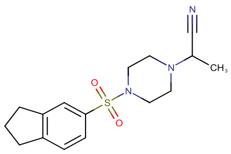	Z52733058	68,65	1,86	++	
724	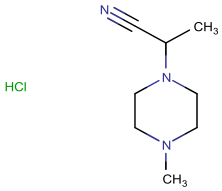	Z54749230	68,47	1,68	++	
725	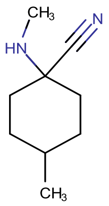	Z56347047	67,11	0,38		
726	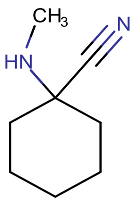	Z56347115	67,29	0,56		
727	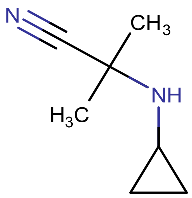	Z56347154	66,55	-0,16		
728	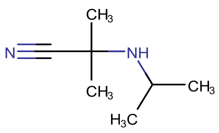	Z56347216	67,02	0,29		

729	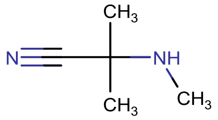	Z56347264	66,55	-0,16		
730	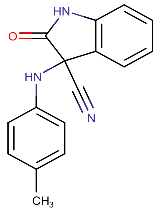	Z56771025	68,20	1,41	++	
731	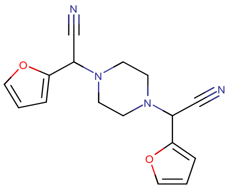	Z56776725	68,92	2,13	++	
732	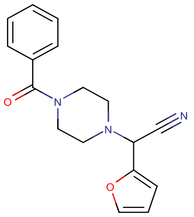	Z56776774	68,65	1,86	++	
733	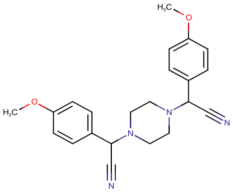	Z56776777	66,85	0,06		
734	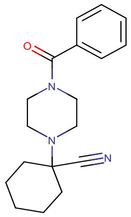	Z56776779	68,38	1,59	++	
735	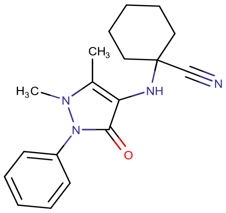	Z56776786	68,20	1,41	++	

736	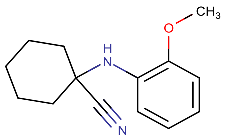	Z56776787	68,20	1,41	++	
737	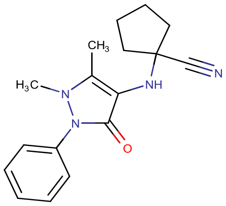	Z56776788	68,65	1,86	++	
738	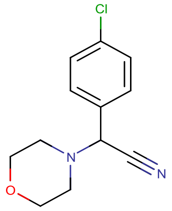	Z56788122	68,11	1,32	++	
739	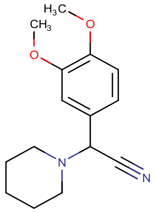	Z56794745	68,65	1,86	++	
740	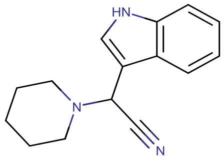	Z56801447	67,57	0,78		
741	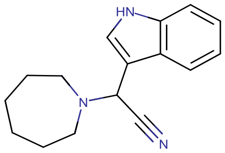	Z56801467	66,94	0,15		
742	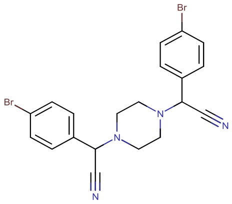	Z56808158	66,94	0,15		

743	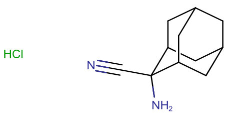	Z56822853	68,65	1,86	++	
744	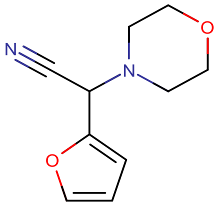	Z56865005	67,83	1,10	++	
745	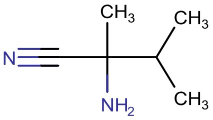	Z56943431	67,02	0,29		
746	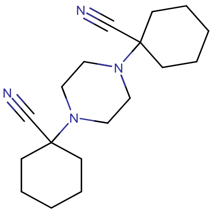	Z56948225	67,57	0,78		
747	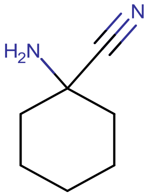	Z56968513	67,83	1,10	++	
748	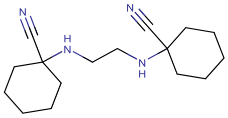	Z56968829	68,29	1,50	++	
749	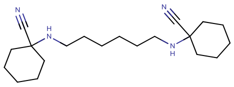	Z56968893	67,93	1,14	++	

750	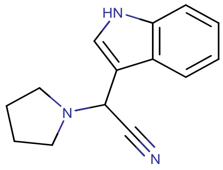	Z57008875	67,21	0,42		
751	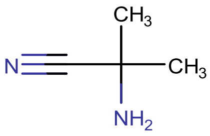	Z57176535	67,65	0,92		
752	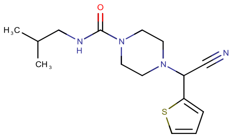	Z642113158	67,84	1,05	++	
753	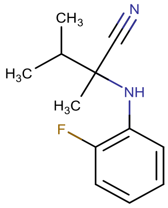	Z727609074	67,11	0,38		
754	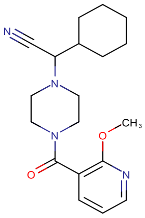	Z729719498	68,11	1,32	++	
755	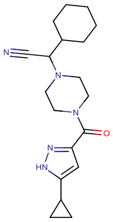	Z729719520	68,20	1,41	++	
756	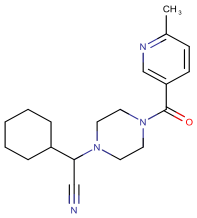	Z729719786	68,65	1,86	++	

757	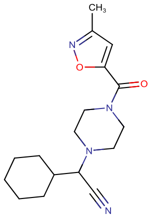	Z729720858	68,11	1,32	++	
758	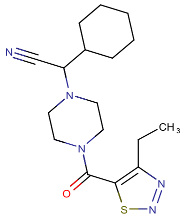	Z729720942	68,38	1,59	++	
759	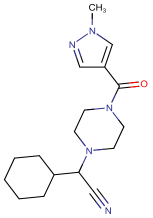	Z729721058	68,11	1,32	++	
760	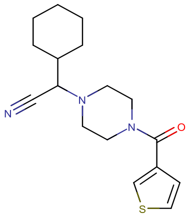	Z729721208	68,56	1,77	++	
761	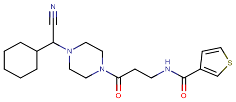	Z729721210	68,02	1,23	++	
762	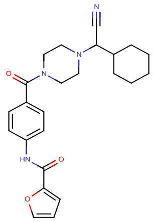	Z729721268	68,56	1,77	++	
763	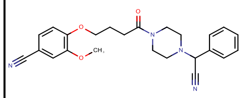	Z743228426	67,36	0,65		

764	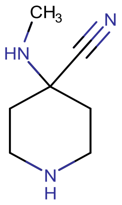	Z778272648	68,55	1,81	++	
765	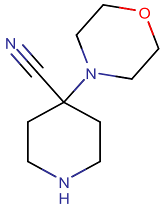	Z778384020	68,44	1,73	++	
766	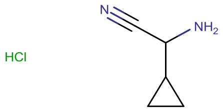	Z802855888	68,29	1,50	++	
767	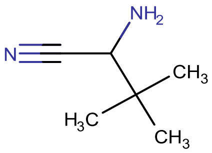	Z803055956	66,91	0,20		
768	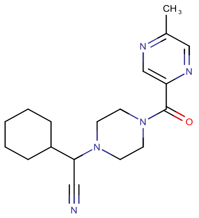	Z816575236	68,38	1,59	++	
769	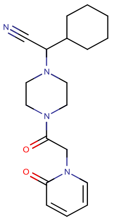	Z816577744	68,02	1,23	++	
770	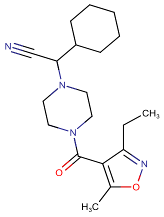	Z816578624	68,38	1,59	++	

771	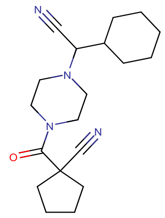	Z816579564	68,20	1,41	++	
772	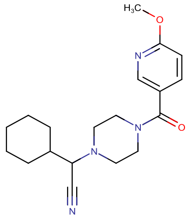	Z816579810	68,47	1,68	++	
773	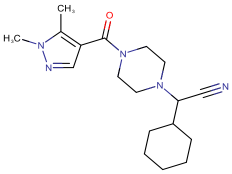	Z816581226	68,02	1,23	++	
774	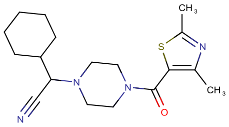	Z816581322	67,30	0,51		
775	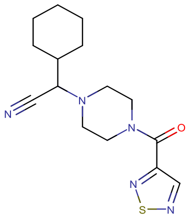	Z816585398	68,20	1,41	++	
776	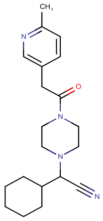	Z816585402	68,47	1,68	++	
777	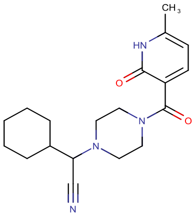	Z816585410	68,38	1,59	++	

778	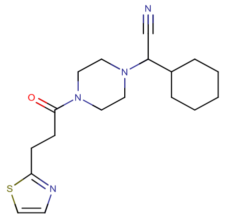	Z816586546	68,11	1,32	++	
779	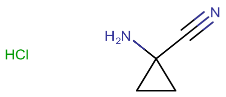	Z826888380	66,55	-0,16		
780	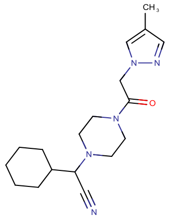	Z849350802	68,38	1,59	++	
781	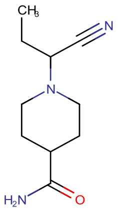	Z851009470	68,53	1,82	++	
782	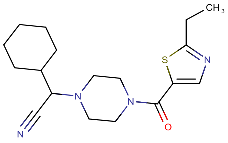	Z855122006	67,18	0,47		
783	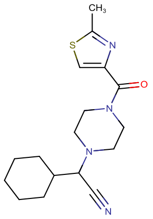	Z878623338	68,11	1,32	++	
784	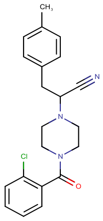	Z89126413	67,03	0,24		

785	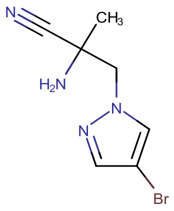	Z898334508	66,94	0,15			
786	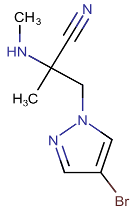	Z898334510	67,39	0,60			
787	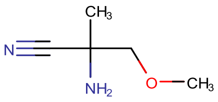	Z898336310	67,92	1,19	++		
788	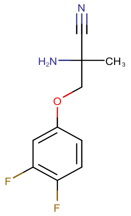	Z898338158	67,92	1,19	++		
789	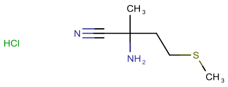	Z898341444	67,54	0,83			
790	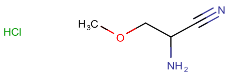	Z898370652	66,64	-0,07			
791	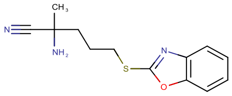	Z898375336	67,56	0,83			

792	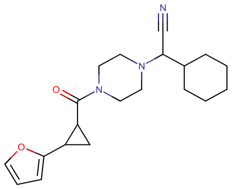	Z899436650	68,56	1,77	++	
793	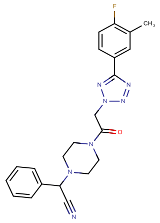	Z912736632	67,18	0,47		
794	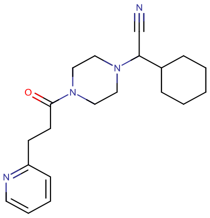	Z913897024	68,56	1,77	++	YES
795	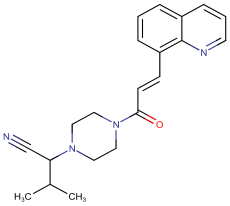	Z991787272	68,29	1,50	++	
796	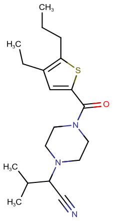	Z991787858	68,47	1,68	++	
797	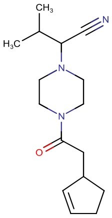	Z991788036	68,56	1,77	++	
798	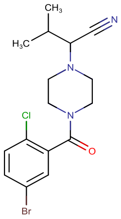	Z991788284	68,56	1,77	++	

799	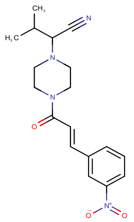	Z991788364	68,38	1,59	++	
800	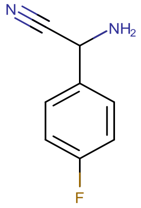	Z99599728	66,75	0,02		
